# Tumor‐Derived Exosomes Deliver Membrane‐Bound Fgl2 to Activate FcγRIIB‐Mediated Immunosuppression in Myeloid‐Derived Suppressor Cells

**DOI:** 10.1002/advs.202521784

**Published:** 2026-03-24

**Authors:** Fenglin Lin, Dingqin Cai, Chunli Jian, Yaxian Qi, Linpeng Zheng, Qiao Yang, Longyao Zhang, Diangang Chen, Lingchen Li, Ping Cai, Lingyou Sun, Luping Zhang, Jianguo Sun

**Affiliations:** ^1^ Department of Oncology, Xinqiao Hospital Army Medical University Chongqing China; ^2^ Department of Hematology and Oncology 921 Hospital of Joint Logistics Support Force of PLA(the Second Affiliated Hospital of Hunan Normal University) Changsha China; ^3^ School of Pharmaceutical Engineering Chongqing Chemical Industry College Chongqing China

**Keywords:** Fgl2‐FcγRIIB axis, immunotherapy, MDSCs, TEX, TME

## Abstract

Myeloid‐derived suppressor cells (MDSCs) play a pivotal role in establishing an immunosuppressive tumor microenvironment (TME), yet the mechanisms underlying their functional activation remain incompletely defined. Here, we identify the Fgl2‐FcγRIIB signaling axis as a critical mediator of MDSC‐driven immune evasion across solid tumors. Analysis of clinical specimens revealed that Fgl2 expression is significantly elevated in tumor tissues and inversely correlates with CD8^+^ T cell infiltration, while positively associating with the accumulation of FcγRIIB^+^ MDSCs and poor patient prognosis. We demonstrate that tumor‐derived exosomes (TEX) function as efficient carriers that deliver membrane‐bound Fgl2 (mFgl2) to MDSCs. These exosomes are internalized by MDSCs through FcγRIIB‐mediated endocytosis, leading to an enhanced immunosuppressive function characterized by upregulated arginase‐1 (Arg‐1) and inducible nitric oxide synthase (iNOS) expression and an increased capacity to suppress CD8^+^ T cell proliferation. Genetic ablation of FcγRIIB or antibody‐mediated neutralization of Fgl2 abolished this exosome‐mediated immunosuppressive programming, restoring T cell activity and impairing tumor growth in vivo. Importantly, a therapeutic strategy combining an exosome secretion inhibitor, in combination with PD‐L1 blockade and MDSCs depletion, synergistically achieved potent antitumor effects. Our findings unveil a novel exosome‐dependent mechanism through which tumors systemically educate MDSCs, establishing the Fgl2‐FcγRIIB axis as a promising broad‐spectrum target for cancer immunotherapy.

## Graphical abstract

This study reveals that the Fgl2‐FcγRIIB signaling axis is a key mechanism by which MDSCs mediate tumor immune evasion. Tumor‐derived exosomes systemically activate MDSCs via this pathway, positioning this axis as a promising broad‐spectrum target for cancer immunotherapy.

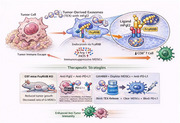

## 1 Introduction

Cancer remains one of the most critical global public health challenges [1], with China reporting the highest number of new cases and death cases worldwide, underscoring the urgent need for novel therapeutic strategies [2, 3]. Although conventional treatments, including chemotherapy, radiotherapy, targeted therapy, and immunotherapy, are widely used, their efficacy remains limited due to cancer cell heterogeneity and TME‐mediated immunosuppression. The efficacy of immunotherapies, particularly immune checkpoint inhibitors (ICIs), is constrained by these immunosuppressive and immune escape mechanisms within the TME [4, 5]. MDSCs are pivotal regulators within this milieu, representing a heterogeneous population of immature myeloid cells with potent immunosuppressive capabilities [6, 7]. Based on phenotype and function, MDSCs are primarily categorized into two subsets: in mice, monocytic MDSCs (M‐MDSCs) are identified as CD11b^+^Ly6G^−^Ly6C^high^, and granulocytic MDSCs (G‐MDSCs) as CD11b^+^Ly6G^+^Ly6C^low^ [8]; in humans, M‐MDSCs are commonly CD11b^+^CD14^+^HLA‐DR^−^/^low^CD15^−^, while G‐MDSCs are CD11b^+^CD14^−^CD15^+^ [9]. MDSCs drive tumor immune escape through multiple mechanisms, including the depletion of essential amino acids (e.g., arginine), production of reactive oxygen and nitrogen species, and induction of T cell apoptosis and exhaustion [10, 11], thereby serving as a key factor in resistance to ICIs therapy (e.g., anti‐PD‐1, anti‐PD‐L1) [12].

Among the myriad molecules regulating MDSCs function, the signaling axis formed by the inhibitory Fc gamma receptor IIB (FcγRIIB, CD32b) and its specific ligand, fibrinogen‐like protein 2 (Fgl2), has garnered increasing interest [13, 14]. FcγRIIB transduces negative regulatory signals via an immunoreceptor tyrosine‐based inhibitory motif (ITIM) in its cytoplasmic tail and has been shown to suppress anti‐tumor immunity in macrophages, dendritic cells(DCs), and T cells [15, 16]. Fgl2, an immunomodulatory protein existing in both membrane‐bound and secreted forms, is highly expressed in various cancers and associated with poor prognosis [17, 18]. Membrane‐bound Fgl2 (mFgl2) is a type II transmembrane protein with a molecular weight of approximately 70 kDa [19, 20]. It contains cytoplasmic, transmembrane, and extracellular domains, and is expressed on the surface of macrophages, T cells, vascular endothelial cells, and various tumor cells as a membrane‐anchored monomer [21, 22]. In contrast, soluble Fgl2 (sFgl2) is secreted primarily by CD4^+^ T cells, CD8^+^ T cells, and regulatory T cells (Tregs). It lacks the transmembrane segment and consists solely of the extracellular domain, forming an approximately 150 kDa tetramer stabilized by disulfide bonds that is released into body fluids such as plasma [23, 24]. These structural and localization differences underlie their distinct biological roles: mFgl2 is overexpressed in multiple tumor types and is implicated in tumor progression [21, 25], whereas sFgl2 functions predominantly as an immunomodulatory molecule [26, 27]. In 2015, Yan et al. established the central role of Fgl2 in glioma, demonstrating that high levels of sFgl2 in the TME promote immunosuppression by driving M2 macrophage polarization, expanding MDSCs, and upregulating immune checkpoint molecules via its receptor, FcγRIIB [28]. Subsequent work by Wu et al. in 2023 refined this understanding by demonstrating that MDSC‐intrinsic Fgl2 enhances their immunosuppressive function via a cell‐intrinsic mechanism that regulates cholesterol metabolism and the XBP1 signaling pathway [29]. In a different approach, Wang et al. in 2024 shifted focus away from targeting sFgl2 itself to developing a peptide vaccine targeting Fgl2. This vaccine induces a specific T‐cell immune response to eliminate Fgl2‐ expressing tumor cells, highlighting the therapeutic potential of targeting the membrane‐bound form [30].

Despite these significant advances, a critical gap and apparent paradox remain in the current understanding of the Fgl2‐FcγRIIB axis. While sFgl2 is considered a diffusible molecule, its stability and targeting efficiency within the complex TME may be limited. Conversely, the mode of action for mFgl2, which has demonstrated clear tumor‐promoting functions, has traditionally been thought to be restricted to the cells that express it. This traditional view fails to adequately explain how tumors can systemically and remotely educate and activate MDSCs, which are dispersed throughout the tumor mass and even in distant lymphoid organs. Furthermore, a key unanswered question remains: whether structural differences between mFgl2 and sFgl2 lead to functional divergence in their regulation of MDSCs.

Exosomes are nanoscale vesicles secreted by cells that deliver bioactive substances such as proteins and nucleic acids, thereby mediating intercellular communication [31, 32]. Notably, the high heterogeneity and physical barriers of the TME limit the diffusion and efficacy of traditional soluble factors. However, exosomes demonstrate significant advantages in transmembrane transport due to their natural membrane fusion capability, immune evasion, ability to traverse physiological barriers, high biocompatibility, engineering potential, and optimal size [33, 34, 35]. Leveraging these properties, exosomes promote immune escape in the TME through various mechanisms. For instance, melanoma‐derived exosomes carry PD‐L1 to directly suppress T cell function [36]; and in liver cancer, GOLM1 promotes PD‐L1 loading into exosomes, thereby inhibiting CD8^+^ T cells [37]. Additionally, in glioblastoma, exosomes activate the AMPK/ULK1 pathway to induce protective autophagy, enhancing chemotherapy resistance [38]. In preliminary transwell co‐culture experiments, we observed significant activation of pathways associated with FcγR‐mediated endocytosis and endocytic vesicle formation in MDSCs, strongly implicating exosome‐mediated signaling in this process. More importantly, we successfully detected specific enrichment of the 70 kDa mFgl2 protein in exosomes derived from the MC38 colon cancer cell line and other tumor cell lines using Western blotting. In contrast, sFgl2 was not detected. Functional assays demonstrated that, compared to exogenous addition of equivalent concentrations of recombinant sFgl2 protein, mFgl2‐loaded exosomes were more potent in upregulating the expression of the immunosuppressive molecules Arg‐1 and inducible iNOS in MDSCs, thereby more robustly enhancing their immunosuppressive capacity.

Our study was designed to resolve this central paradox. Diverging from prior research focused on sFgl2 protein or Fgl2 peptide vaccines, we identified a novel signaling paradigm mediated by TEX. We discovered that tumor cells efficiently load mFgl2 onto the surface of exosomes. These mFgl2‐bearing exosomes function as a precise “Trojan horse” delivery system, effectively overcoming the spatial limitations traditionally associated with mFgl2 action and enabling the remote regulation of MDSCs. Furthermore, we elucidated that MDSCs actively internalize these exosomes via their surface FcγRIIB receptor. This internalization triggers a distinct and potent intracellular immunosuppressive signaling cascade, different from the pathway activated by sFgl2‐FcγRIIB interaction, which leads to the marked upregulation of key immunosuppressive molecules such as Arg‐1 and iNOS. Consequently, our work not only uncovers a novel exosome‐mFgl2‐FcγRIIB regulatory pathway for MDSCs, challenging the conventional understanding of mFgl2's functional scope, but also establishes a remote communication mechanism in tumor immunosuppression that is more efficient and stable than signaling by soluble molecules. These findings provide a solid theoretical foundation for developing broad‐spectrum immunotherapeutic strategies targeting this novel communication system.

## 2 Results

### 2.1 Spatial Interaction of the Fgl2‐FcγRIIB Axis Between Tumor Cells and MDSCs Drives Immuno‐Suppression and Disease Progression in Colorectal Cancer

**FIGURE 1:**
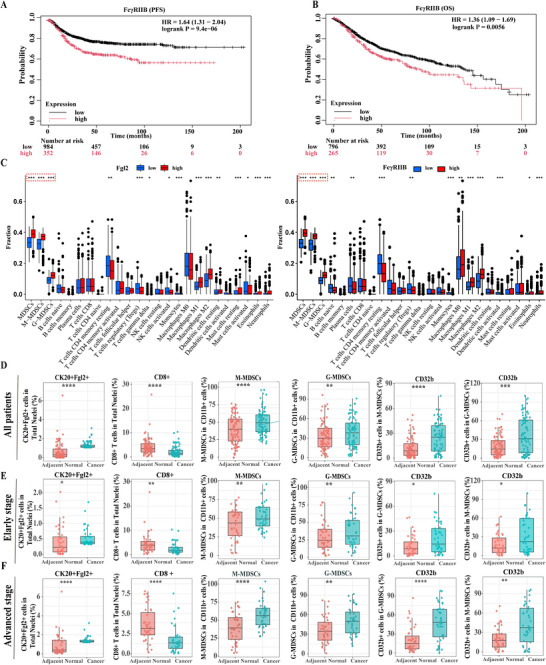
Spatial profiling of Fgl2–FcγRIIB axis and immune contexture in human colorectal cancer. (A‐B) Association between FcγRIIB gene expression levels and overall survival (OS) or progression‐free survival (PFS) in colon cancer patients. (C) Infiltration levels of 25 immune cell types in groups with high versus low expression of Fgl2 and FcγRIIB. (D‐F) Comparisons between colon cancer tissues and adjacent normal tissues regarding the proportion of FGL2^+^ tumor cells, CD8^+^ T cell density, M‐MDSCs (CD11b^+^CD14^+^)/G‐MDSCs (CD11b^+^CD15^+^) ratio, and FcγRIIB expression in MDSCs subsets: (D) all patients; (E) early‐stage (I‐II); (F) late‐stage (III‐IV). Data are presented as box plots overlaid with scatter plots, where boxes display the median (center line), interquartile range (box boundaries), and range (whiskers); individual data points are shown as overlaid dots. Kaplan‐Meier curves were analyzed by the log‐rank test. Statistical comparisons between tumor and adjacent normal tissue data were performed using the Wilcoxon matched‐pairs signed‐rank test (^*^ *p* < 0.05, ^**^ *p* < 0.01, ^***^ *p* < 0.001, ^****^ *p* < 0.0001).

Patients in the TCGA colon adenocarcinoma (COAD) cohort were stratified into an FcγRIIB‐high (n = 352) and an FcγRIIB‐low (n = 796) group based on the median expression level of FcγRIIB. Prognostic analysis, encompassing overall survival (OS) and progression‐free survival (PFS), revealed that elevated FcγRIIB expression was significantly associated with adverse clinical outcomes. Kaplan‐Meier curves demonstrated that the FcγRIIB‐high group had significantly shorter OS (Log‐rank p = 0.0056; Figure 1B) and PFS (Log‐rank p = 9.4e‐06; Figure 1A) compared to the low‐expression group. This was corroborated by univariate Cox regression analysis, which identified high FcγRIIB expression as an independent risk factor for both OS (HR = 1.36, 95% CI: 1.09–1.69, p = 0.0056) and PFS (HR = 1.64, 95% CI: 1.31–2.04, p = 9.4e‐06). These data indicate that FcγRIIB not only correlates with poor survival but is also implicated in early disease progression and recurrence, highlighting its potential as a robust prognostic biomarker in COAD.

To investigate the impact on the tumor immune microenvironment, we evaluated the correlation between Fgl2/FcγRIIB expression and the infiltration levels of 25 immune cell types in the TCGA‐COAD cohort. The expression of both genes significantly reshaped the immune landscape. We focused on MDSCs and their subsets due to their potent immunosuppressive functions. Notably, high expression of Fgl2 and FcγRIIB was significantly positively correlated with increased infiltration of total MDSCs, as well as M‐MDSCs and G‐MDSCs subsets (Figure 1C). This suggests that the Fgl2‐FcγRIIB axis may foster an immunosuppressive niche in colon cancer, potentially by recruiting or expanding MDSCs.

**FIGURE 2:**
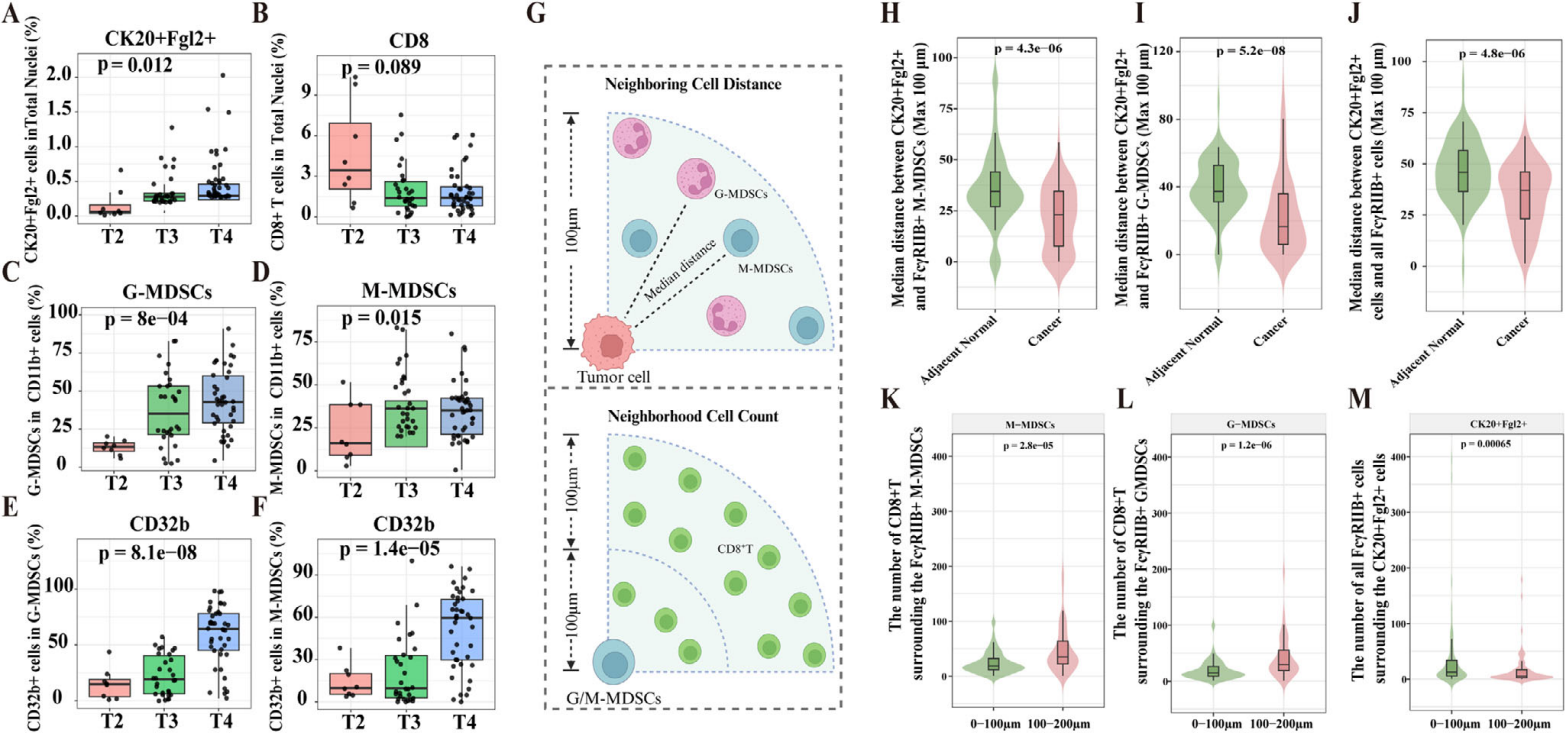
Spatial distribution and cellular interactions in the colon cancer microenvironment. (A–F) Comparisons between tumor and adjacent tissues across T stages regarding CK20+ Fgl2^+^ tumor cell proportion, CD8^+^ T cell density, M‐MDSCs (CD11b^+^CD14^+^)/G‐MDSCs (CD11b^+^CD15^+)^ ratio, and FcγRIIB expression on MDSCs. (G) Schematic of intercellular distance measurement and neighborhood analysis, which was created with BioRender.com, for which a publication license has been obtained. (H) Mean distance between CK20^+^FGL2^+^ tumor cells and FcγRIIB+ M‐MDSCs in tumor vs. normal tissues. (I) Mean distance between CK20^+^FGL2^+^ cells and FcγRIIB+ G‐MDSCs. (J) Mean distance between CK20^+^FGL2^+^ cancer cells and all FcγRIIB^+^ cells. (K) CD8^+^ T cell distribution at varying distances from FcγRIIB^+^ M‐MDSCs. (L) CD8^+^ T cell distribution around FcγRIIB^+^ G‐MDSCs. (M) All FcγRIIB^+^ cell enrichment near CK20^+^FGL2^+^ tumor cells. (A–F) Box plots display the median (center line), interquartile range (box boundaries), and range (whiskers). (H–M) Violin plots combined with box plots. (A–F) Group differences were assessed by the Kruskal–Wallis test, followed by Dunn's post‐hoc test with Bonferroni correction for multiple comparisons where appropriate; (H–J) tumor–normal comparisons used Wilcoxon matched‐pairs signed‐rank test; (K–M) used Mann‐Whitney U test for unpaired comparisons. ^*^ *p* < 0.05, ^**^ *p* < 0.01, ^***^ *p* < 0.001, ^****^ *p* < 0.0001.

To gain initial insight into the functional role of the Fgl2‐FcγRIIB axis in modulating MDSCs activity, we performed multiplex immunofluorescence on a tissue microarray comprising 80 colon cancer patients (markers: CK20, Fgl2, FcγRIIB, CD11b, CD14, CD15, CD8; Figure S1A) [39]. In tumor tissues, we observed significant upregulation of Fgl2, increased infiltration of FcγRIIB^+^ MDSCs, and a concomitant reduction in CD8^+^ T cells compared to matched adjacent normal tissues (Figure 1D). This pattern was exacerbated in advanced‐stage (Stage III/IV) compared to early‐stage (Stage I/II) diseases, with further elevation of Fgl2, MDSCs infiltration, and MDSCs FcγRIIB expression, alongside a pronounced decrease in CD8^+^ T cells (Figure 1E,F). A similar trend was observed during local tumor progression (T2 to T4 stage), where all three parameters increased progressively while CD8^+^ T cell counts declined (Figure 2A,F). These findings strongly associate Fgl2‐FcγRIIB axis activation with disease progression and immunosuppression. In contrast, this signature showed no significant association with lymph node metastasis (N stage), except for a minor increase in MDSCs FcγRIIB in N1‐N2 disease (Figure S1B), suggesting a primary role in local immune modulation rather than lymphatic spread. Analysis of distant metastasis (M stage) revealed a slight upregulation of FcγRIIB on MDSCs in metastatic cases (Figure S1C), implying a potential link between this axis and systemic immunosuppression supporting metastasis, which warrants further investigation as a therapeutic target.

We employed neighborhood cell‐cell distance analysis (defined as the average distance between a target cell and its neighboring cells within a specified 100 µm radius) and neighborhood cell counting (quantifying neighboring cells within 0–100 µm and 100–200 µm radii from target cells) to investigate the spatial distribution and potential interactions of different cell populations within tissues(Figure 2G) [40, 41, 42].

Our analysis revealed that in tumor tissues, the average spatial distances between CK20^+^Fgl2^+^ tumor cells and both FcγRIIB^+^ M‐MDSCs and FcγRIIB^+^ G‐MDSCs were significantly shorter compared to adjacent normal tissues (Figure 2H,I), suggesting closer interactions between tumor cells and FcγRIIB^+^ MDSCs within the TME. Similarly, the average distance between CK20^+^Fgl2^+^ tumor cells and cells expressing FcγRIIB was markedly reduced in tumor tissues (Figure 2J), further supporting potential specific interactions between Fgl2 and FcγRIIB. Furthermore, when analyzing the spatial distribution of CD8^+^ T cells around FcγRIIB^+^ M‐MDSCs and FcγRIIB^+^ G‐MDSCs, we observed significantly higher numbers of CD8^+^ T cells in the 100–200 µm range compared to the immediate 0–100 µm neighborhood (Figure 2K,L). This spatial pattern suggests that FcγRIIB^+^ MDSCs may establish an immunosuppressive microenvironment that excludes or inhibits the infiltration and function of CD8^+^ T cells. Conversely, neighborhood analysis centered on CK20^+^Fgl2^+^ tumor cells demonstrated higher enrichment of cells expressing FcγRIIB in closer proximity to tumor cells (Figure 2M). This spatial configuration aligns with the expected pattern of Fgl2‐FcγRIIB ligand‐receptor interaction and indicates the functional relevance of this pathway within the TME.

### 2.2 Spatiotemporal FcγRIIB Expression in MDSCs Correlates with Immunosuppressive Function

Although previous clinical data indicated elevated FcγRIIB expression on MDSCs in colon cancer, correlating with disease progression, the inherent limitations of retrospective clinical data preclude definitive conclusions regarding its direct regulatory role in MDSCs differentiation and function. MDSCs, originating from bone marrow progenitors, are broadly classified into the monocytic (M‐MDSCs) and granulocytic (G‐MDSCs) subsets [43]. While tumor‐derived factors (e.g., GM‐CSF, IL‐6) are known to drive MDSCs' expansion, how specific receptor signaling influences their subtype differentiation remains unclear. Previous studies suggest FcγRIIB can negatively regulate Toll‐like receptor signaling in antigen‐presenting cells, suppressing their activation and inhibiting pro‐inflammatory cytokine (e.g., IL‐12) secretion [44]. Furthermore, specific signals within the tumor TME, such as IL‐6/TGF‐β or exosomal miRNAs, may dynamically modulate FcγRIIB expression via epigenetic modifications (e.g., promoter methylation) or transcription factors (e.g., NF‐κB), consequently influencing MDSCs functional plasticity [45].

**FIGURE 3:**
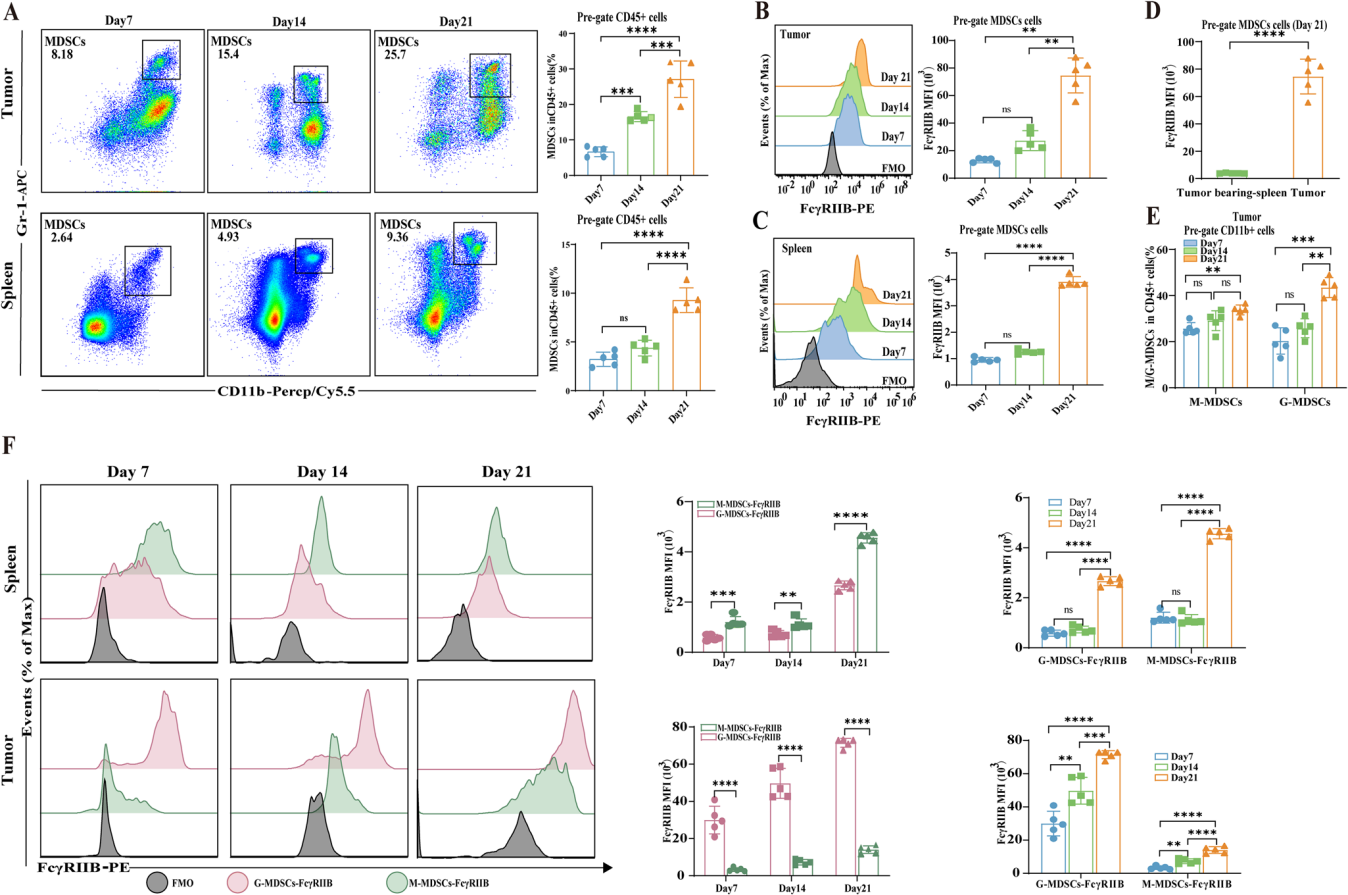
Dynamic expression patterns of FcγRIIB in MDSCs and correlation with immunosuppressive function. (A) Representative flow cytometry plots and quantitation of MDSCs (CD11b^+^Gr‐1^+^) proportions in tumor tissues and spleen during tumor progression (days 7/14/21). (B) Flow cytometry histograms and MFI showing temporal changes in FcγRIIB expression on tumor‐infiltrating MDSCs. (C)Flow cytometry histograms and MFI showing temporal changes in FcγRIIB expression on splenic MDSCs. (D) Statistical bar graph showing FcγRIIB expression levels in tumor versus splenic MDSCs at day 21. (E) Temporal dynamics of the proportions of M‐MDSCs (CD11b+Ly6c^high^) and G‐MDSCs (CD11b+Ly6g^high^) subsets within tumor‐infiltrating MDSCs, presented as a statistical bar graph. (F) Statistical bar graphs showing the temporal dynamics (Day 7, 14, 21) of FcγRIIB expression (MFI) in MDSCs subsets from the spleen and tumor tissue, as determined by flow cytometry. All data were acquired by flow cytometry (n = 5). Statistical comparisons were performed using one‐way ANOVA with Tukey's HSD post‐hoc test. Data presented as mean ± SEM. ^*^ *p* < 0.05, ^**^ *p* < 0.01, ^***^ *p* < 0.001, ^****^ *p* < 0.0001.

To delineate the dynamic regulation of FcγRIIB in MDSCs‐mediated immunosuppression, we established a murine subcutaneous transplant tumor model and systematically analyzed MDSCs distribution and FcγRIIB expression in both tumors and spleens at different stages of progression (Day 7, 14, and 21). Flow cytometry analysis revealed a significant time‐dependent increase in the proportion of MDSCs within both tumor tissues and spleens (Figure 3A). FcγRIIB expression on tumor‐infiltrating MDSCs increased progressively over time (Figure 3B), consistent with the trend observed in splenic MDSCs (Figure 3C). Tissue‐specific analysis demonstrated that FcγRIIB expression on MDSCs was significantly higher in the TME compared to their splenic counterparts (Figure 3D), suggesting local microenvironmental factors drive its upregulation. Subset analysis showed synchronous expansion of both M‐MDSCs and G‐MDSCs in late‐stage tumors (Figure 3E).

Notably, we observed a distinct pattern of FcγRIIB expression on MDSCs subsets between tumor and splenic tissues. In the spleens of tumor‐bearing mice, M‐MDSCs exhibited higher FcγRIIB expression than G‐MDSCs. However, upon migration into the tumor tissue, G‐MDSCs significantly upregulated FcγRIIB, reaching levels approximately 3.5‐fold higher than those on M‐MDSCs (Figure 3F). Overall, FcγRIIB expression on both M‐MDSCs and G‐MDSCs increased over time in both compartments.

This study elucidates the dynamic, tissue microenvironment‐dependent expression pattern of FcγRIIB during tumor progression. Its spatiotemporal upregulation is closely associated with MDSCs‐mediated immunosuppression. The time‐dependent increase of FcγRIIB on tumor‐infiltrating MDSCs aligns with our findings in the human colon cancer tissue microarray. These results identify FcγRIIB as a potential biomarker for MDSCs functional activation and provide a rationale for staging‐specific, FcγRIIB‐targeted precision immunotherapy strategies.

### 2.3 FcγRIIB Deficiency Reshapes the Immunosuppressive Microenvironment and Enhances Antitumor Immunity

**FIGURE 4:**
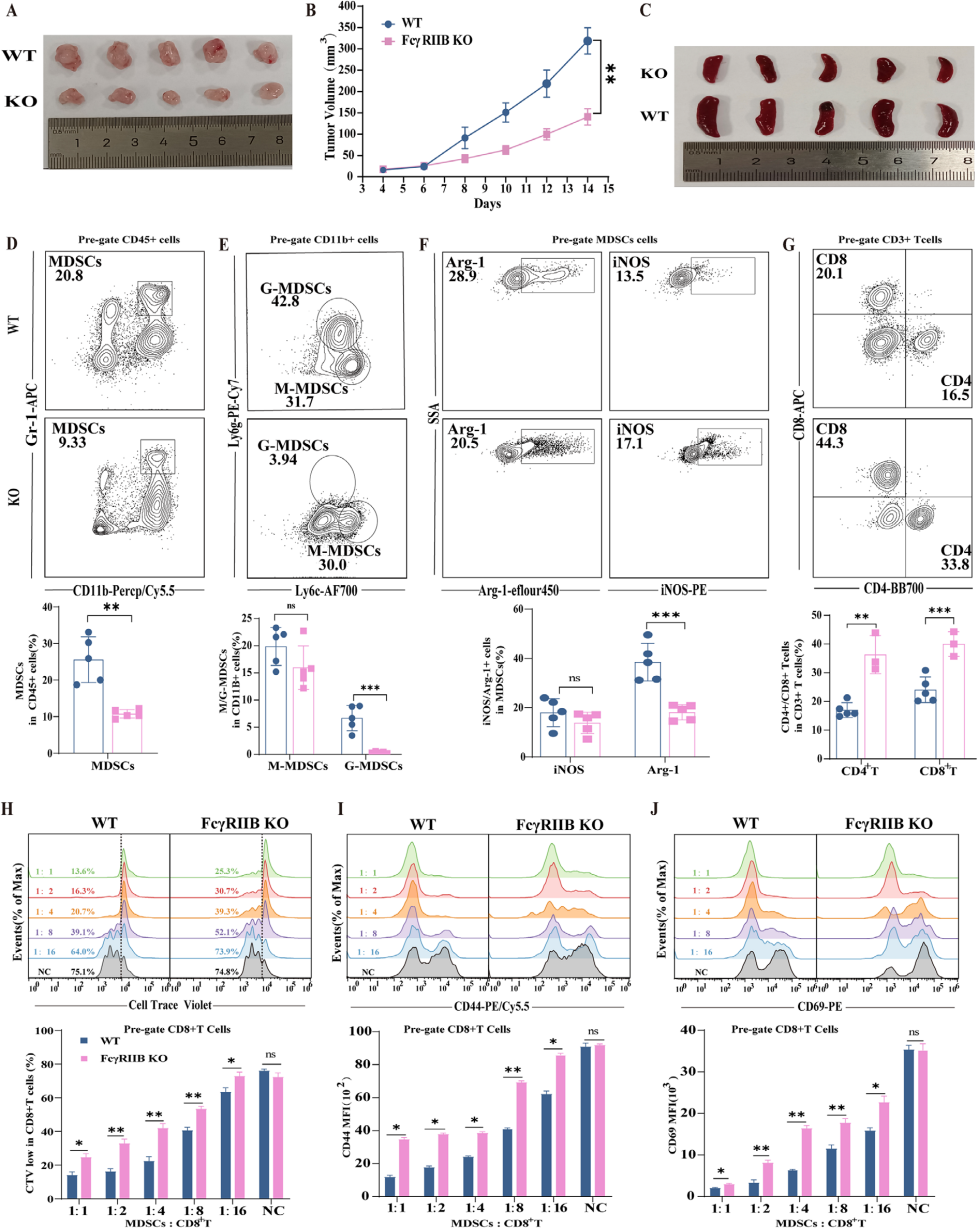
FcγRIIB influences antitumor immune responses by regulating MDSC function. (A) Gross images of tumors at the experimental endpoint. (B) Tumor growth curves in WT and FcγRIIB KO mice. (C) Gross images of spleens at the experimental endpoint. (D) Representative flow cytometry contour plots and statistical bar graphs showing the proportion of MDSCs infiltrating tumor tissues. (E) Representative flow cytometry contour plots and statistical bar graphs showing the proportions of M‐MDSC and G‐MDSC subsets in tumor tissues. (F) Representative flow cytometry contour plots and statistical bar graphs showing Arg‐1 and iNOS expression in tumor‐infiltrating MDSCs. (G) Representative flow cytometry contour plots and statistical bar graphs showing infiltration of CD4^+^ and CD8^+^ T cells within tumors. (H) Representative flow cytometry CTV proliferation peaks and statistical bar graphs showing CD8^+^ T cell proliferation after co‐culture with WT or FcγRIIB KO MDSCs. (I‐J) Representative flow cytometry histograms and statistical bar graphs showing expression of CD44 and CD69 on CD8^+^ T cells. For in vivo flow cytometry data, n = 5; for in vitro experiments, n = 3. Statistical analysis: comparisons between two groups were performed using Student's t‐test; comparisons among three or more groups were performed using one‐way ANOVA followed by Tukey's HSD post‐hoc test. Data are presented as mean ± SEM. ^*^ *p* < 0.05, ^**^ *p* < 0.01, ^***^ *p* < 0.001, ^****^ *p* < 0.0001.

FcγRIIB, an inhibitory Fc gamma receptor, plays a dual role in tumor immune regulation. FcγRIIB is expressed on immune cells within the tumor microenvironment, including MDSCs, CD8+ T cells, tumor‐associated macrophages (TAMs), and dendritic cells [14, 29, 46, 47]. It can competitively bind the Fc portion of IgG antibodies, thereby inhibiting Antibody‐Dependent Cellular Cytotoxicity/Phagocytosis (ADCC/ADCP) and impairing the efficacy of therapeutic antibodies [15]. Conversely, it mediates immunosuppression by promoting M2 macrophage polarization, suppressing M1 polarization, and inhibiting the NF‐κB signaling pathway [48, 49]. To further investigate the regulatory role of FcγRIIB on MDSCs function, we established a subcutaneous transplant tumor model using FcγRIIAB‐knockout (KO) mice, confirmed as homozygotes by PCR. Experimental results demonstrated that compared to wild‐type (WT) mice, FcγRIIB‐KO tumor‐bearing mice exhibited significantly slower tumor growth (Figure 4A,B) and no splenomegaly (Figure 4C), suggesting that FcγRIIB deficiency systemically inhibits tumor progression and alleviates associated splenic immunopathology.

Comparative flow cytometric analysis of the TME and splenic immune cell composition in FcγRIIB‐KO versus WT tumor‐bearing mice revealed that FcγRIIB deletion significantly reshaped both local and systemic anti‐tumor immune responses. Within tumor tissues, the KO group showed reduced total MDSCs infiltration (Figure 4D). Notably, the proportion of the highly immunosuppressive G‐MDSCs subset decreased, while the proportion of M‐MDSCs among MDSCs increased relatively (Figure 4E). Furthermore, Arg‐1 expression was significantly downregulated in KO mice (Figure 4F), indicating that FcγRIIB enhances immunosuppression by promoting G‐MDSCs differentiation and activating the Arg‐1 pathway. Concurrently, the macrophage population increased with a shift toward enhanced M1 polarization (Figure S2A), suggesting improved antigen presentation and pro‐inflammatory immune activation. Dendritic cells (DCs) in KO mice also showed elevated expression of the co‐stimulatory molecule CD86 (Figure S2B). Accordingly, tumor infiltration by both CD4^+^ and CD8^+^ T cells was significantly increased in KO mice (Figure 4G). Consistent changes were observed in the spleen: total MDSCs, including both G‐MDSCs and M‐MDSCs subsets, were reduced (Figure S2C,D), alongside downregulated expression of Arg‐1 and iNOS (Figure S2E). The proportions and activation states of DCs and M1 macrophages were elevated (Figure S2G,H), and the splenic ratios of CD4^+^ and CD8^+^ T cells were significantly higher than in WT controls (Figure S2F). These results demonstrate that FcγRIIB deficiency not only locally ameliorates the tumor immune microenvironment but also systemically impairs MDSCs‐mediated immunosuppression, thereby promoting anti‐tumor T cell responses. Our study reveals that FcγRIIB coordinately sustains an immunosuppressive state at both local and systemic levels by regulating macrophage polarization, G‐MDSCs differentiation, and immunosuppressive molecule expression, positioning FcγRIIB as a promising target for enhancing anti‐tumor immunity.

To definitively establish the role of FcγRIIB in regulating the immunosuppressive function of MDSCs, we performed further validation via in vitro co‐culture experiments. When magnetically sorted MDSCs were co‐cultured with CD8^+^ T cells, MDSCs derived from FcγRIIB‐KO mice exhibited a significantly diminished capacity to suppress CD8^+^ T cell proliferation compared to their WT counterparts (Figure 4H). Consequently, CD8^+^ T cells in KO co‐cultures expressed higher levels of activation markers CD44 and CD69 (Figure 4I,J), indicating that FcγRIIB deficiency impairs MDSCs‐mediated T cell suppression. This in vitro finding aligns with the in vivo phenotype, suggesting FcγRIIB cell‐autonomously enhances the immunosuppressive function of MDSCs.

Building on the spatiotemporal expression disparity of FcγRIIB across MDSCs subsets‐notably high on G‐MDSCs in tumor tissue and relatively higher on M‐MDSCs in the spleen (Figure 3F), and the observed G‐MDSCs developmental defect in KO mice, we investigated its regulatory mechanism on subset differentiation using a bone marrow cell in vitro differentiation model. Flow cytometric analysis revealed a lower proportion of MDSCs generated from FcγRIIB‐KO mouse‐derived bone marrow cells compared to the WT group (Figure S2I). Subset analysis further identified a predominance of M‐MDSCs (approximately 35%) in the KO group, while the G‐MDSCs proportion was about 20% lower than in the WT group (p < 0.01), where G‐MDSCs were the dominant subset (Figure S2J). This in vitro differentiation phenotype, highly consistent with the MDSCs subset distribution observed in the in vivo tumor model, indicates that FcγRIIB not only influences MDSCs quantity but also directly steers their differentiation toward the G‐MDSCs subset, thereby potentiating their immunosuppressive function.

### 2.4 Targeting the mFgl2‐FcγRIIB Axis Reverses MDSCs‐Mediated Immunosuppression and Synergizes with PD‐L1 Blockade

**FIGURE 5:**
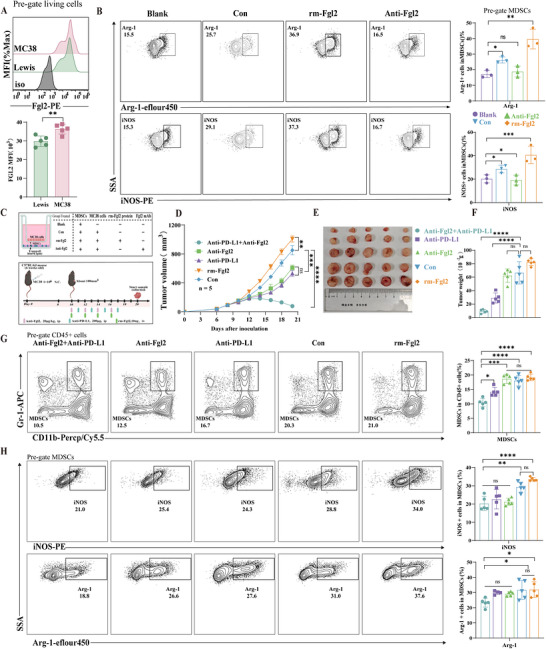
Fgl2 regulates MDSCs function and influences immunotherapy response via FcγRIIB. (A) Representative flow cytometry histograms and statistical bar graphs showing Fgl2 expression in MC38 and Lewis cells. (B) Representative flow cytometry contour plots and statistical bar graphs showing Arg‐1 and iNOS expression in MDSCs after non‐contact co‐culture with tumor cells under different treatment conditions. (C) Schematic illustration of the in vitro experimental groups and in vivo treatment regimen, created with BioRender.com, for which a publication license has been obtained. (D) Tumor growth curves. (E) Gross images of tumors at the experimental endpoint. (F) Tumor weights at the experimental endpoint. (G‐H) Representative flow cytometry contour plots and statistical bar graphs showing infiltration of MDSCs and their expression of iNOS and Arg‐1 in tumor tissues. All flow cytometry data were obtained from n = 5 mice per group. Statistical comparisons between two groups were performed using an unpaired Student's t‐test. Comparisons among three or more groups were performed using one‐way ANOVA followed by Tukey's HSD post‐hoc test. Data are presented as mean ± SEM. Significance is defined as ^*^ *p* < 0.05, ^**^ *p* < 0.01, ^***^ *p* < 0.001, ^****^ *p* < 0.0001.

Fgl2, a multifunctional protein expressed in both membrane‐bound and secreted forms [18, 50], engages inhibitory signaling pathways via receptors including FcγRIIB, and plays a pivotal role in shaping the tumor immune microenvironment [13, 17, 51]. Elevated Fgl2 expression correlates with poor prognosis across multiple malignancies [52]. Western blotting analysis confirmed high mFgl2 expression in MC38, Lewis, and Hepa1‐6 cell lines (Figure S3A), with flow cytometry further validating stable expression in MC38 and Lewis cells (Figure 5A). Using a non‐contact co‐culture system (Figure 5C), we demonstrated that MC38 cells enhance the immunosuppressive function of MDSCs via soluble factors‐including Fgl2‐as indicated by upregulated Arg‐1 and iNOS expression (Figure 5B). This effect was augmented by exogenous recombinant sFgl2 (20 ng/mL) and partially reversed using an mFgl2‐blocking antibody, suggesting cooperative regulation of MDSCs activity by additional tumor‐derived factors.

To determine FcγRIIB dependency, we treated MDSCs from wild‐type (WT), FcγRIIB‐knockout (KO), and FcγRIIB‐low genotypes with sFgl2. High FcγRIIB‐expressing WT MDSCs showed significantly elevated Arg‐1 and iNOS following 20 ng/mL sFgl2 stimulation, with no further increase at higher concentrations (40–100 ng/mL), indicating receptor saturation (Figure S3C). In contrast, low‐FcγRIIB MDSCs were unresponsive even at high sFgl2 doses (Figure S3D), and FcγRIIB‐KO MDSCs completely lost functional responsiveness (Figure S3E), confirming the essential and non‐redundant role of FcγRIIB in Fgl2‐mediated immunosuppression.

For in vivo validation, we established MC38 subcutaneous tumor models under five conditions: (1) control; (2) rmFgl2 infusion; (3) anti‐mFgl2; (4) anti‐PD‐L1; (5) anti‐mFgl2 + anti‐PD‐L1. Tumor growth was significantly suppressed in the combination group (Figure 5C–F). Flow cytometry revealed reduced MDSCs infiltration and suppressed iNOS/Arg‐1 expression in tumors from the combination group (Figure 5G,H). Anti‐PD‐L1 monotherapy reduced MDSCs frequency but not suppressor function, whereas rmFgl2 enhanced iNOS. Concurrently, combination treatment increased tumor‐infiltrating CD8^+^ T cells and enhanced IFN‐γ and TNF‐α production in both CD4^+^ and CD8^+^ T cells (Figure S4A,D). Splenic analysis revealed elevated MDSCs frequency only with rmFgl2 (Figure S4E). While iNOS remained unchanged across groups, Arg‐1 was elevated in rmFgl2 groups(Figure S3F,G), suggesting the immunosuppressive activity of splenic MDSCs was enhanced in a tumor‐bearing state. T cell function in the spleen was maintained in the combination group despite possible effector T cell migration to tumors, whereas rmFgl2 suppressed cytokine production (Figure S3G,H).

Our findings illustrate the organ‐specific modulation of immune responses via the Fgl2‐FcγRIIB axis. Combined inhibition reduces local MDSCs infiltration and function while promoting T cell activation and trafficking, supporting a multimodal strategy to alleviate immunosuppression. Further investigation is required to dissect the downstream mechanisms and cellular crosstalk underlying this effect.

### 2.5 Exosomal mFgl2 is a Potent Activator of MDSCs Immunosuppression via FcγRIIB‐Mediated Uptake

The TME constitutes a highly complex and dynamic ecosystem whose immunosuppressive properties largely rely on sophisticated intercellular communication networks [53, 54, 55, 56]. Beyond traditional soluble factors such as cytokines and chemokines, exosomes‐nanoscale vesicles measuring 30–150 nm in diameter‐act as “molecular containers” that efficiently deliver bioactive molecules, including proteins and nucleic acids, offering unique advantages in mediating signaling within the TME [31, 32, 57, 58]. Using a non‐contact co‐culture system of MC38 tumor cells and MDSCs, combined with transcriptomic sequencing, we observed significant enrichment of pathways related to receptor‐mediated endocytosis, notably Fc gamma receptor (FcγR)‐mediated endocytosis and the Fgl2‐FcγRIIB signaling axis. Western blotting further confirmed specific expression of mFgl2 in MC38‐derived exosomes, suggesting that tumor cells may actively enrich mFgl2 into exosomes, thereby enabling targeted delivery.

Although exosome‐mediated transfer of immunosuppressive molecules such as TGF‐β or PD‐L1 has been reported, the role of mFgl2 as an exosomal cargo protein remains unclear. Based on these findings, we hypothesize that tumor cells may employ exosomes to deliver mFgl2 and remotely regulate the immunosuppressive function of MDSCs.

**FIGURE 6:**
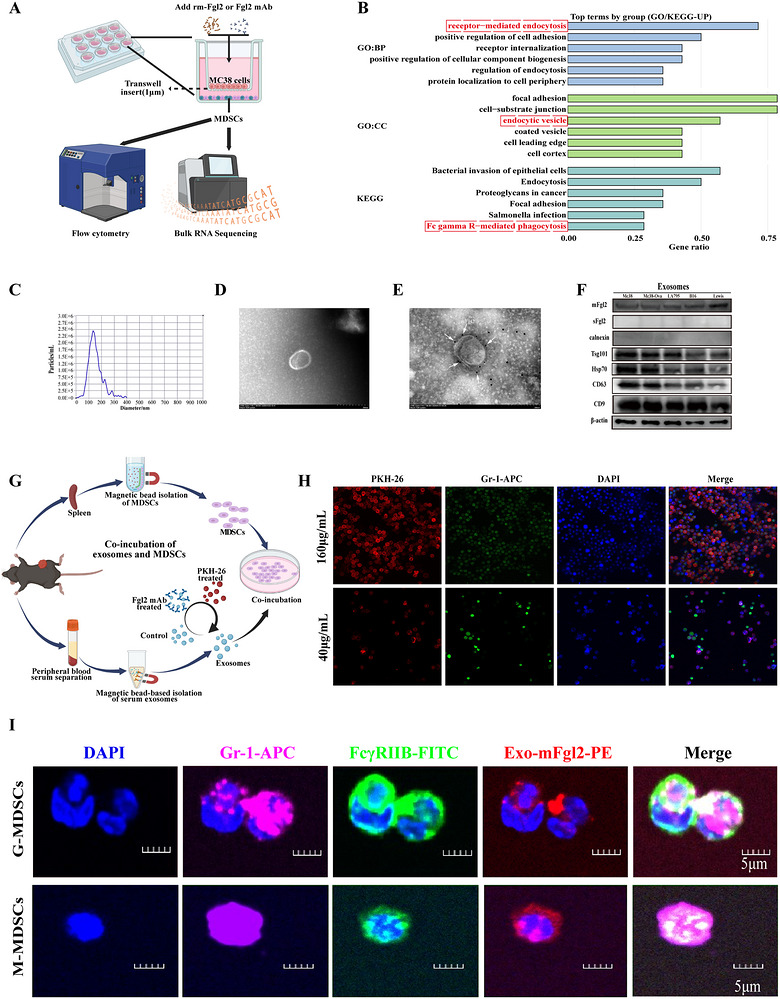
Mechanistic study of exosomal Fgl2 enhancing the immunosuppressive function of MDSCs via FcγRIIB. (A) Schematic diagram of non‐contact co‐culture between tumor cells and MDSCs. (B) Transcriptome enrichment analysis of pathways, including receptor‐mediated endocytosis and FcγR‐mediated phagocytosis in the co‐culture group compared to MDSCs cultured alone. (C) Nanoparticle tracking analysis of tumor cell‐derived exosomes. (D) Transmission electron microscopy image of tumor cell‐derived exosomes. (E) Immunogold electron microscopy image of mFgl2 on tumor cell‐derived exosomes. (F) Western blot characterization of tumor cell‐derived exosomes. (G) Schematic diagram of co‐incubation of mouse peripheral blood‐derived exosomes with MDSCs. (H) Tracing of PKH26‐labeled tumor exosomes after co‐incubation with MDSCs. (I) Confocal microscopy imaging showing colocalization of fluorescently labeled tumor exosomal mFgl2 with FcγRIIB on the surface of MDSCs after co‐incubation.

Functional assays revealed that both a co‐culture system and exogenous sFgl2 protein potentiate the immunosuppressive activity of MDSCs. Transcriptomic profiling comparing MDSCs cultured alone versus those co‐cultured with tumor cells demonstrated significant enrichment of pathways governing receptor‐mediated endocytosis, receptor internalization, and Fc gama R‐mediated phagocytosis (Gene Ontology and Kyoto Encyclopedia of Genes and Genomes analyses; p < 0.05). These findings raised the possibility that exosomes might engage FcγRIIB through Fgl2 to initiate internalization (Figure 6A,B). Subsequent investigations substantiated this hypothesis. Exosomes isolated from tumor cell supernatants exhibited characteristic features validated by electron microscopy, nanoparticle tracking analysis (NTA), and western blotting (Figure 6C,D), and were found to harbor mFgl2 protein (Figure 6F). Immunoelectron microscopy further confirmed that mFgl2 on the exosomal surface retained its structural integrity and bioactivity, as evidenced by the decoration with colloidal gold particles (Figure 6E).

Tracking experiments utilizing PKH26‐labeled exosomes demonstrated their efficient internalization by MDSCs, with fluorescent signals translocating from the plasma membrane to the cytoplasm and perinuclear region over time (Figure 6G,H). This uptake correlated with accelerated MDSCs proliferation. To directly visualize the interaction between exosomal mFgl2 and MDSCs surface FcγRIIB, exosomes captured on magnetic beads were surface‐labeled for mFgl2 and incubated with MDSCs. Subsequent immunostaining for Gr‐1 and FcγRIIB, followed by confocal microscopy, revealed distinct colocalization of mFgl2 with FcγRIIB (Figure 6I), providing direct evidence of their molecular interaction.

**FIGURE 7:**
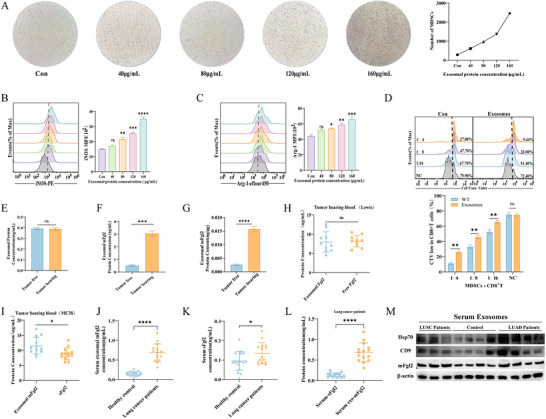
Exosomal mFgl2 enhances MDSCs immunosuppressive function and is elevated in the serum of tumor‐bearing mice and lung cancer patients. (A) Visual representation and linear trend of MDSCs proliferation status after co‐incubation with tumor‐derived exosomes at different protein concentrations. (B‐C) Representative flow cytometry histograms and statistical bar graphs showing iNOS and Arg‐1 expression in MDSCs. (D) Representative flow cytometry CTV proliferation peaks and statistical bar graphs showing CD8^+^ T cell proliferation after co‐culture with MDSCs pre‐treated with tumor‐derived exosomes. (E) Statistical bar graph showing total protein quantification of serum exosomes from tumor‐bearing C57BL/6J mice (Day 14) versus healthy mice. (F‐G) Statistical bar graphs showing mFgl2 levels in serum exosomes from tumor‐bearing C57BL/6J mice (Day 14) versus healthy mice, quantified by ELISA. (H‐I) Statistical bar graphs comparing exosomal mFgl2 and serum sFgl2 levels in Lewis and MC38 tumor‐bearing mice, quantified by ELISA. (J) Statistical bar graphs comparing serum exosomal mFgl2 and sFgl2 levels between lung cancer patients(n = 5) and healthy volunteers(n = 6), quantified by ELISA. (L) Statistical bar graph comparing serum exosomal mFgl2 and sFgl2 levels in lung cancer patients(n = 5), quantified by ELISA. (M) Western blot detection of serum exosomal mFgl2 and sFgl2 in lung cancer patients (n = 7) versus healthy volunteers(n = 4). Each experiment was independently repeated three times. Statistical comparisons between two groups were performed using an unpaired Student's t‐test. Comparisons among three or more groups were performed using one‐way ANOVA followed by Tukey's HSD post‐hoc test. Data are presented as mean ± SEM. Significance is defined as ^*^ *p* < 0.05, ^**^ *p* < 0.01, ^***^ *p* < 0.001, ^****^ *p* < 0.0001.

Functionally, tumor‐derived exosomes promoted MDSCs expansion and upregulated the expression of immunosuppressive effectors, including Arg‐1 and iNOS, in a concentration‐dependent fashion (Figure 7A,C). In co‐cultures with CD8^+^ T cells, MDSCs pre‐exposed to tumor exosomes exhibited a markedly enhanced capacity to suppress T cell proliferation, an effect proportional to the MDSCs‐to‐T cell ratio (Figure 7D).

To delineate the relative contributions of exosome‐associated mFgl2 versus soluble Fgl2 (sFgl2), we first isolated exosomes from the serum of Lewis lung carcinoma‐bearing mice (day 14) and healthy controls. While total exosomal protein content was comparable between groups (Figure 7E,F), the concentration of mFgl2 was approximately sixfold higher in the tumor‐bearing cohort, quantified at 0.0158 ng/µg exosomal protein compared to 0.0026 ng/µg in healthy controls (Figure 7G). This suggested that circulating exosomal mFgl2 in tumor‐bearing hosts is predominantly tumor‐derived. A comparative analysis in Lewis and MC38 models revealed that exosomal mFgl2 levels were comparable to sFgl2 in Lewis mice, but markedly higher in MC38 mice, with calculated mFgl2 cargo loads of 0.018 and 0.028 ng/µg for Lewis and MC38 exosomes, respectively (Figure 7H,I). Translating these findings to human disease, exosomes isolated from the plasma of lung cancer patients (n = 5) contained significantly higher levels of both mFgl2 and sFgl2 compared to healthy donors (n = 6)(Figure 7J,K). Notably, within the patient group, the concentration of exosome‐associated mFgl2 exceeded that of sFgl2(Figure 7L). This observation was corroborated by western blot analysis of exosomes from lung squamous cell carcinoma (n = 3), lung adenocarcinoma (n = 4), and healthy donors (n = 4), collectively indicating a marked elevation of exosomal mFgl2 in cancer patients(Figure 7M).

**FIGURE 8:**
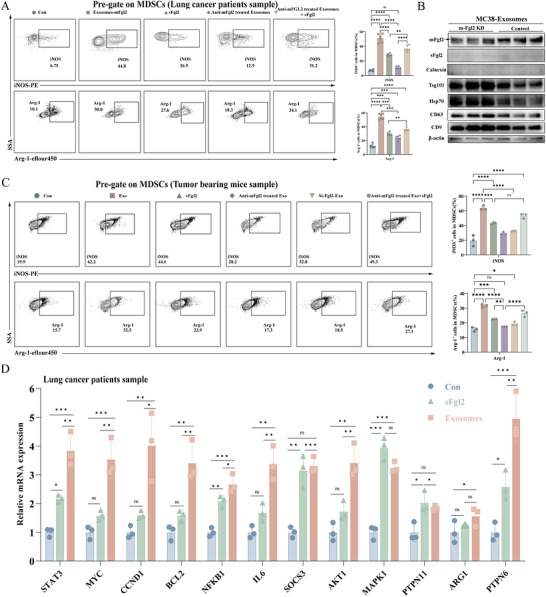
Exosomal mFgl2 directly activates human and mouse MDSCs and engages the ITIM signaling pathway. (A) Schematic of experimental workflow: exosomes and PBMCs were isolated from the peripheral blood of lung cancer patients. Exosomes were quantified for mFgl2 concentration by ELISA and total protein by BCA assay. PBMCs were expanded into MDSCs in vitro using hGM‐CSF (40 ng/mL) and hIL‐6 (40 ng/mL) for 5 days. Exosomal mFgl2 was adjusted to the physiological concentration detected in lung cancer patient plasma (0.75 ng/mL) and co‐incubated with MDSCs in complete medium at 37°C for 12 h. Human sFgl2 was used at the same concentration as a control. Flow cytometry was performed to detect iNOS and Arg‐1 expression in MDSCs. Representative flow cytometry contour plots and statistical bar graphs showing iNOS and Arg‐1 expression in MDSCs across different treatment groups (n = 5). (B) Western blot analysis of mFgl2 expression in exosomes derived from si‐Fgl2 MC38 tumor cells and controls. (C) Exosomes from MC38 tumor‐bearing mice were quantified and adjusted to 10 ng/mL mFgl2, then co‐incubated with MDSCs in complete medium at 37°C for 12 h. Mouse sFgl2 was used at the same concentration as a control. Representative flow cytometry contour plots and statistical bar graphs showing iNOS and Arg‐1 expression in MDSCs (n = 3). (D) Lung cancer patient‐derived exosomes were quantified for mFgl2 and compared with sFgl2 at equivalent concentrations. After 12 h of co‐culture with MDSCs, mRNA expression levels of downstream signaling molecules in the FcγRIIB ITIM pathway were detected by qPCR. Each experiment was independently repeated three times. Comparisons among three or more groups were performed using one‐way ANOVA followed by Tukey's HSD post‐hoc test. Data are presented as mean ± SEM. Significance is defined as ^*^ *p* < 0.05, ^**^ *p* < 0.01, ^***^ *p* < 0.001, ^****^ *p* < 0.0001.

The specific functional relevance of exosomal mFgl2 was interrogated using MDSCs expanded ex vivo from lung cancer patient peripheral blood mononuclear cells. Although recombinant sFgl2 potentiated MDSCs suppressive activity, exosome‐associated mFgl2 at an equivalent concentration exerted a more profound effect, eliciting higher iNOS and Arg‐1 expression. Pre‐incubation of exosomes with an mFgl2‐neutralizing antibody abrogated this enhanced suppression, reducing MDSCs function to near‐baseline levels. Importantly, this inhibitory effect was partially rescued by the addition of exogenous sFgl2 (Figure 8A), indicating that while exosomal mFgl2 is a primary driver, sFgl2 can also contribute to the regulatory milieu. To exclude antibody off‐target effects, we employed MC38 cells stably expressing Fgl2‐targeting shRNA (si‐Fgl2), which produced exosomes with markedly reduced mFgl2 cargo (Figure 8B). These mFgl2‐deficient exosomes exhibited a significantly impaired capacity to induce MDSCs suppressive function compared to wild‐type TEX (Figure 8C), mirroring the antibody blockade results. Collectively, these data establish that exosomes serve as a highly effective delivery system for mFgl2, which potentiates MDSCs function more robustly than its soluble counterpart, highlighting exosomal mFgl2 as a potential immunotherapeutic target.

Mechanistically, engagement of FcγRIIB by Fgl2 is known to trigger signaling via the receptor's intracellular immunoreceptor tyrosine‐based inhibitory motif (ITIM). Phosphorylated ITIM recruits SH2 domain‐containing phosphatases, including SHP‐1(PTPN6), SHP‐2(PTPN11), SHIP‐1(INPP5D), which subsequently activate transcription factors driving iNOS and Arg‐1 expression, often in coordination with STAT3 signaling [59, 60]. Quantitative PCR analysis of MDSCs following stimulation revealed that while both exosomal and soluble Fgl2 induced mRNA expression of ITIM pathway components, the exosomal form elicited a more potent transcriptional response, with higher expression levels of most downstream effectors (Figure 8D). This provides a molecular basis for the enhanced functional outcomes observed with exosomal mFgl2.

In conclusion, this study integrates multi‐omics analysis, imaging‐based validation, functional assays, and signaling pathway exploration to elucidate a mechanism wherein tumor‐derived exosomes deliver mFgl2 to MDSCs. This engagement, mediated by FcγRIIB, results in exosome internalization and augments MDSCs immunosuppressive activity. The specific abrogation of this effect by mFgl2‐targeting antibodies not only confirms pathway specificity but also furnishes a preclinical rationale for therapeutic strategies aimed at neutralizing exosomal mFgl2 to alleviate immunosuppression and potentially enhance the efficacy of existing immunotherapies.

### 2.6 Triple Therapy Co‐Targeting Exosomes and MDSCs Elicits Potent Antitumor Immunity

**FIGURE 9:**
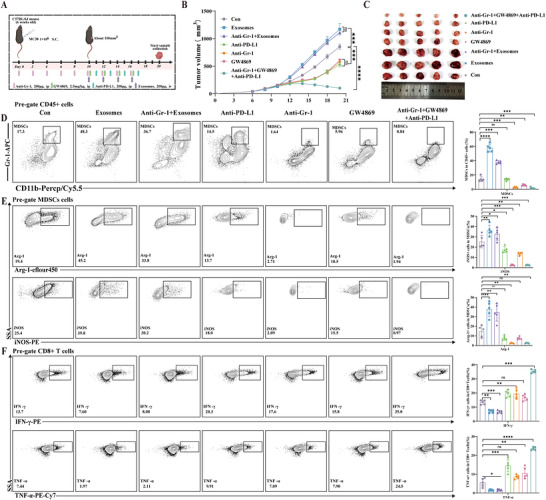
Triple therapy co‐targeting exosomes and MDSCs overcomes immunosuppression. (A) Schematic of the animal treatment protocol, created with BioRender.com and used under license. Treatment regimen: Starting at day 10 (tumor volume ∼100 mm^3^), mice received i.p. injections every 2 days for 4 doses of anti‐PD‐L1 (200 µg/dose), GW4869 (2.5 mg/kg), or PBS (control). MDSC depletion was performed with anti‐Gr‐1 antibody (200 µg/dose i.p. every 2 days for 9 doses) beginning at tumor inoculation. Adoptive transfer of MC38 tumor‐derived exosomes (200 µg exosomal protein/dose) was administered i.v. Every 2 days for 4 doses from day 10. (B) Tumor growth curves across different treatment groups. (C) Tumor weight at the experimental endpoint. (D) Representative flow cytometry contour plots and percentages of tumor‐infiltrating MDSCs. (E) Flow cytometry contour plots and percentages of Arg‐1 and iNOS expression in tumor‐infiltrating MDSCs. (F) Proportions of CD4^+^ and CD8^+^ T cells in tumor tissues. Data were acquired by flow cytometry (n=5). Statistical comparisons were performed using one‐way ANOVA with Tukey's HSD post‐hoc test. Data are presented as mean ± SEM. ^*^ *p* < 0.05, ^**^ *p* < 0.01, ^***^ *p* < 0.001, ^****^ *p* < 0.0001.

Using a murine subcutaneous transplant tumor model, animals were divided into the following treatment groups: (1) control; (2) exosome reinfusion; (3) MDSCs depletion + exosome reinfusion; (4) anti‐PD‐L1 monoclonal antibody; (5) MDSCs depletion; (6) exosome inhibitor (GW4869); and (7) triple therapy with MDSCs depletion + GW4869 + anti‐PD‐L1. MDSCs depletion began on the day of tumor inoculation, and treatments or reinfusion of tumor‐derived exosomes were initiated when tumors reached approximately 100 mm^3^ on day 10 (Figure 9A). Results showed that exosome reinfusion significantly promoted tumor growth, while monotherapies (anti‐PD‐L1, anti‐Gr‐1, or GW4869) only mildly delayed tumor progression. Notably, the triple therapy group exhibited markedly suppressed tumor growth, with regression observed after day 14 (Figure 9B,C).

Further mechanistic investigations revealed that reinfusion of mFgl2‐positive exosomes substantially increased MDSCs infiltration within the TME (Figure 9D), enhanced their immunosuppressive function (Figure 9E), reduced the proportions of CD4^+^ and CD8^+^ T cells, and induced functional exhaustion of CD8^+^ T cells (Figure 9F; Figure S4K). Additionally, macrophages showed decreased M1 polarization and increased M2 polarization (Figure S4M,N), indicating that exosomes may regulate myeloid immunity through a dual mechanism: promoting MDSCs accumulation and impairing antitumor macrophage polarization.

In mice subjected to MDSCs depletion followed by exosome reinfusion, MDSCs proportions remained higher than those in the control group (Figure 9D), and their immunosuppressive function was not significantly compromised (Figure 9E). This suggests that exosomes not only recruit MDSCs but may also directly maintain their immunosuppressive phenotype through epigenetic reprogramming or paracrine signaling, and might even induce in situ differentiation of myeloid precursors into MDSCs [61, 62, 63, 64]. In this group, T cell proportion and function remained severely suppressed (Figure 9F; Figure S4K), and macrophages were polarized toward an M2 phenotype (Figure S4N).

In contrast, the triple therapy group exhibited synergistic antitumor effects at multiple levels: GW4869‐mediated inhibition of exosome secretion blocked Fgl2 delivery and nearly completely eliminated MDSCs in the TME (Figure 9D). This was accompanied by increased CD8^+^ T cell infiltration, enhanced production of IFN‐γ and TNF‐α (Figure 9F; Figure S4K), elevated dendritic cell proportions with upregulated CD86 expression (Figure S4L), and a shift in macrophage polarization toward M1 and away from M2 (Figure S4N). Ultimately, these changes fostered an immune‐activated microenvironment that significantly suppressed tumor growth.

Meanwhile, we further examined the infiltration of MDSCs and CD8^+^ T cells in tumor tissues by immunofluorescence staining. The results were consistent with our earlier flow cytometry findings (Figure S5F).

To determine whether the reduced MDSCs infiltration in the GW4869‐treated group in the animal experiments was caused by the inhibition of mFgl2‐carrying exosome secretion, we treated MC38 and Lewis cells with the exosome inhibitor GW4869 (30 µM) and isolated exosomes from the cell culture supernatant. We then measured the total protein concentration of the exosomes using the BCA assay, and assessed mFgl2 protein expression by ELISA and Western blot. The results showed that after GW4869 treatment, the secretion of exosomes from tumor cells was significantly suppressed, as indicated by a decrease in total protein concentration, and the level of mFgl2 protein carried by the exosomes was also markedly reduced. This indicates that GW4869 effectively inhibits tumor exosome secretion from MC38 and Lewis cells, thereby reducing the release of mFgl2 (Figure S5A,B).

Subsequently, we isolated MDSCs from the spleens of tumor‐bearing C57BL/6J mice and labeled them with CellTrace Violet (CTV). These labeled MDSCs were then co‐cultured with MC38 and Lewis tumor cells in a transwell system (Figure S5C), while the lower chamber containing tumor cells was treated with GW4869 (30 µM). After 24 h of co‐culture, the lower chamber of the 12‐well plate was imaged under a fluorescence microscope to observe the migration of CTV^+^ MDSCs (Figure S5D). Finally, the migrated MDSCs in the lower chamber were analyzed by flow cytometry, and their absolute numbers were quantified. The results showed that treatment of tumor cells with GW4869 resulted in a reduction of the absolute number of migrating MDSCs compared to the control group (Figure S5E).

In summary, mFgl2‐positive exosomes regulate the immune microenvironment through exosomal mFgl2, which exhibits a stronger capacity to activate MDSCs compared to its soluble form: first by recruiting MDSCs, then enhancing their immunosuppressive function and inhibiting M1 macrophage polarization. Even under conditions of MDSCs deficiency, exosomes maintain immunosuppression through as‐yet‐unknown pathways. This study innovatively reveals that exosomes modulate MDSCs via a three‐phase “recruitment‐reinforcement‐maintenance” model and demonstrates that combined targeting of the exosome‐MDSCs axis and PD‐L1 blockade effectively reverses immunosuppression, providing a novel strategy and theoretical foundation for cancer immunotherapy.

## 3 Discussion

In the current study, we integrated multiple approaches, including tissue microarray analysis of clinical samples, FcγRIIB knockout mouse models, in vitro co‐culture systems, exosome manipulation techniques, and multi‐omics analysis. This work identifies a previously unrecognized mechanism of immunosuppression in which tumor cell‐derived exosomes serve as carriers for mFgl2, which activates the inhibitory receptor FcγRIIB on MDSCs, thereby potentiating their immunosuppressive function and accelerating tumor progression. The characterization of this “exosomal mFgl2‐FcγRIIB” axis provides mechanistic insight into how tumors exploit extracellular vesicles to modulate the immune microenvironment, revealing a potential target for combinatorial immunotherapy. Analysis of clinical specimens initially revealed a positive correlation between elevated Fgl2 expression in colorectal tumor tissues, infiltration of MDSCs, and upregulation of FcγRIIB, a relationship most pronounced during early‐stage local invasion (T staging), suggesting involvement in initial immune escape.

These findings integrate two previously distinct research themes, the immunosuppressive role of mFgl2 and the regulatory function of FcγRIIB, and establish MDSCs as a critical effector population downstream of FcγRIIB signaling, consistent with its documented inhibitory effects in TAMs and Tregs. For example, in TAMs, sFgl2 binds to the FcγRIIB receptor on CD8^+^ T cells, triggering caspase‐dependent apoptosis and thereby suppressing anti‐tumor immunity [52]. In glioblastoma models, combining anti‐PD‐1 therapy with FcγRIIB blockade significantly enhances CD8^+^ T cell function [65]; FcγRIIB upregulation on TAMs correlates with poor prognosis, and anti‐Fgl2 therapy prolongs survival in an FcγRIIB‐dependent manner by reducing immunosuppressive cells. Furthermore, hypoxic signaling via HIF‐1α upregulates FcγRIIB expression in TAMs, promoting their M2 polarization [66]. Conversely, inhibiting FcγRIIB reprograms TAMs toward a pro‐inflammatory phenotype, highlighting its potential as an immunomodulatory target [67]. In melanoma, FcγRIIB on CD8^+^ T cells drives an autocrine Fgl2 apoptotic loop, while TAM‐derived Fgl2 directly kills FcγRIIB^+^ CD8^+^ T cells; FcγRIIB deletion enhances antitumor immunity. In NSCLC, host Fgl2 deficiency suppresses tumor growth via MDSCs and CAFs modulation. Collectively, these data establish the Fgl2‐FcγRIIB axis as a conserved immunosuppressive mechanism across multiple malignancies, supporting its potential as a broad‐spectrum immunotherapeutic target.

Our clinical data revealed a balanced distribution of pMMR (46.25%) and dMMR (53.75%) CRC patients, prompting us to examine the relationship between the Fgl2‐FcγRIIB axis and microsatellite instability (MSI) status. While dMMR tumors typically exhibit high immunogenicity, they often develop adaptive resistance mechanisms [68, 69]. Notably, mFgl2 expression inversely correlated with CD8^+^ T cell infiltration and positively with FcγRIIB^+^ MDSCs accumulation [29, 70]. Given that dMMR patients generally respond better to immune checkpoint blockade, this axis may contribute to immune evasion in dMMR tumors that fail immunotherapy [71, 72]. Conversely, in immunologically “cold” pMMR tumors, it might represent a dominant immunosuppressive pathway amenable to therapeutic targeting [73, 74]. The functional impact of this axis likely extends beyond direct MDSCs activation to influence their differentiation fate. Fgl2 deficiency promotes MDSCs maturation and reduces G‐MDSCs accumulation via cholesterol metabolism regulation [18], while FcγRIIB signaling through Sp1/Stat3 drives MDSCs commitment [29] and sustains their abundance [28]. Given STAT3's established role in MDSCs differentiation [75, 76], this axis probably intersects with known pathways to shape MDSCsplasticity within distinct microenvironments—potentially explaining the differential immune landscapes of pMMR and dMMR tumors. Future studies stratified by MSI status are warranted to determine whether Fgl2 blockade differentially synergizes with immunotherapies and to dissect its crosstalk with microenvironmental regulators such as GM‐CSF and HIF‐1α [77, 78].

The novelty of this work lies in three key advances: first, delineating the dynamic expression profile of FcγRIIB across MDSCs subsets and establishing its specific regulatory role in G‐MDSCs differentiation; second, uncovering a previously unrecognized mechanism whereby tumor‐derived mFgl2, delivered via exosomes, orchestrates MDSCs functional polarization through a dual mode of “membrane signal activation and cargo delivery”. Our results extend prior reports of exosomal miRNA‐ or cytokine‐mediated MDSCs modulation by illustrating a protein ligand‐mediated pathway capable of precise immune regulation [79, 80, 81, 82]; and third, demonstrating the synergistic efficacy of targeting this axis—using mFgl2‐neutralizing antibodies or exosome inhibitors—in combination with anti‐PD‐L1 therapy to reverse immunosuppression and suppress tumor growth. Notably, the functional superiority of exosomal mFgl2 over its soluble counterpart is likely attributable to multiple factors. As a type II transmembrane protein presented as a monomer on exosome surfaces—in contrast to the tetrameric configuration of secreted sFgl2—mFgl2 may exhibit distinct receptor‐binding properties that enhance signaling efficiency. Furthermore, encapsulation within exosomes likely safeguards mFgl2 from proteolytic degradation in the tumor microenvironment, thereby prolonging its bioavailability for FcγRIIB engagement on MDSCs. Compounding these advantages, exosomes possess an intrinsic capacity to traverse physiological barriers and fuse with target cells, potentially facilitating more efficient delivery of mFgl2 to MDSCs. Collectively, these findings not only provide a fresh perspective on the complex intercellular communication networks within the tumor microenvironment but also establish a robust theoretical and experimental foundation for developing novel combination immunotherapies targeting the FcγRIIB‐mediated myeloid checkpoint.

Several limitations merit consideration. The associations identified between FcγRIIB expression on MDSCs and nodal/distant metastatic stages warrant cautious interpretation given the limited cohort size, particularly for M1 disease (n = 11). The marginal increase observed in N1‐N2 cases may reflect a predominant role of this axis in local immune modulation rather than lymphatic dissemination, whereas the modest upregulation in metastatic patients hints at a potential contribution to systemic immunosuppression. Notably, the reversal in expression hierarchy between M‐MDSCs and G‐MDSCs when comparing the spleen and tumor site underscores a profound influence of the local microenvironment on FcγRIIB regulation. in vivo administration of tumor‐derived exosomes rendered MDSCs resistant to Gr‐1 antibody‐mediated depletion; paradoxically, this treatment not only failed to ablate MDSCs but instead elevated their proportion. Functional validation was performed using Fgl2‐knockdown rather than CRISPR/Cas9‐mediated knockout cells, which may not fully eliminate potential off‐target effects or residual protein function. While the mechanisms underlying mFgl2 packaging into tumor‐derived exosomes remain unclear. The downstream signaling pathways through which mFgl2^+^ exosomes upregulate Arg‐1 and iNOS in MDSCs remain to be fully elucidated.

In summary, we propose that tumor‐derived exosomes encapsulate mFgl2 to engage FcγRIIB on MDSCs in paracrine fashion, amplifying immunosuppression and fostering immune evasion. These findings underscore the potential of targeting the exosomal Fgl2‐FcγRIIB axis‐possibly in conjunction with existing immunotherapies‐to disrupt immunosuppressive networks and enhance antitumor immunity.

## 4 Materials and Methods

### 4.1 Data Analysis

Data were analyzed using the following methods: Flow cytometry data were processed using Flow Jo software (BD, version 10). The grayscale values of Western blotting bands were quantified using ImageJ software (NIH). BCA protein quantification and ELISA concentrations were calculated based on OD values obtained from the microplate reader. Statistical analyses and graph generation were performed primarily in R language (version 4.2.0) and GraphPad Prism 8 (GraphPad Software, Inc.). Continuous variables are presented as median with interquartile range or mean ± standard error of the mean (SEM), as appropriate based on data distribution characteristics. For data following a normal distribution (verified by Shapiro‐Wilk test), parametric tests were applied: unpaired t‐test for comparisons between two groups; one‐way or two‐way analysis of variance (ANOVA) for multi‐group comparisons, followed by Tukey's or Šídák's multiple comparisons test for post‐hoc analysis if main effects were significant. For skewed (non‐normal) distribution or non‐parametric data (e.g., most TMA indicators): paired comparisons (e.g., tumor vs. adjacent tissue) used the Wilcoxon signed‐rank test; comparisons among multiple independent groups (e.g., T stage, N stage) used the Kruskal‐Wallis test; comparisons between two independent unpaired groups used the Mann‐Whitney U test. Detailed statistical test information is provided in the respective figure legends. Significance levels were denoted as follows: ^*^ *p* < 0.05, ^**^ *p* < 0.01, ^***^ *p* < 0.001, ^****^ *p* < 0.0001.

## Author Contributions

F.L. designed the study, performed cellular and animal experiments, and completed the first draft. D.C. and C.J. analyzed data, carried out exosome isolation, flow cytometry, and Western blotting. Q.Y. and L.L. conducted the bioinformatics analysis and R language‐based figure generation. L.Z. and Q.Y. assisted with cell culture and functional assays. L.Z. conducted animal experiments. D.C. and Y.Q. performed Western blotting and sample processing. L.S. and P.C. provided technical support. L.Z. performed exosome isolation, characterization, and labeling. J.S. supervised the research. J.S. revised the manuscript, acquired funding and approved the final version. All authors reviewed and approved the manuscript.

## Funding

This work was supported by the Noncommunicable Chronic Diseases‐National Science and Technology Major Project (2023ZD0502100 to J. S.), the National Natural Science Foundation of China (82473261, 82172670 and 81602688 to J.S. and L.Z., respectively), and the Chongqing Medical Leading Talent Project (YXLJ202405 to J.S.).

## Conflicts of Interest

The authors declare no conflicts of interest.

## Supporting information




**Supporting File**: advs74862‐sup‐0001‐SuppMat.docx

## Data Availability

The data that support the findings of this study are available in the supplementary material of this article.
