# Supplementary material for: Tumor‐Derived Exosomes Deliver Membrane‐Bound Fgl2 to Activate FcγRIIB‐Mediated Immunosuppression in Myeloid‐Derived Suppressor Cells
Source: Adv Sci (Weinh). 2026 Mar 24;13(35):e21784. doi: 10.1002/advs.202521784 (PMC13292161; doi:10.1002/advs.202521784)
Supplement: Supplementary file 1 — Supporting File: advs74862‐sup‐0001‐SuppMat.docx [file ADVS-13-e21784-s001.docx]

**Supporting Information：**

**Tumor-Derived Exosomes Deliver Membrane-bound Fgl2 to Activate FcγRIIB-Mediated Immunosuppression in Myeloid-Derived Suppressor Cells**

Fenglin Lin^1#^, Dingqin Cai^2#^, Chunli Jian1^#^, Yaxian Qi^1^, Linpeng Zheng^1^, Qiao Yang^1^, Longyao Zhang^1^, Diangang Chen^1^, Lingchen Li^1^, Ping Cai^1^, Lingyou Sun^1^, Luping Zhang^3^, Jianguo Sun^1*^

**Materials**

**Table S1:** **Lists of reagent or sources**

| **Material** | **Brand** | **Cat#** | **Clone#** |
| --- | --- | --- | --- |
| 100 cm² Cell Culture Dish | Corning | 430167 | / |
| 15 mL Centrifuge Tube | LABSLECT | CT-002-15A | / |
| 1.5 mL Microcentrifuge Tube | Corning | MCT-150-C | / |
| 3 mL Pasteur Pipette | NEST | 318314 | / |
| 5 mL Flow Cytometry Tube | Falcon | 352008 | / |
| 50 mL Centrifuge Tube | LABSLECT | CT-022-50A | / |
| 6-well Cell Culture Plate | Corning | 3516 | / |
| 70 μm Cell Strainer | Falcon | 352350 | / |
| Cell Counting Chamber Slide | Count Star | 12-0005-50 | / |
| Disposable Insulin Syringe | BD | 328421 | / |
| Sterile Disposable Syringe Filter (0.22 μm) | Merck Millipore | SLGPR33RB | / |
| Sterile Disposable Syringe Filter (0.45 μm) | Merck Millipore | SLHPR33RB | / |
| T25 Cell Culture Flask | Corning | 430639 | / |
| T75 Cell Culture Flask | Corning | 430641 | / |
| Ultrafiltration Tube (15 mL, 30 kDa) | Merck Millipore | UFC903096 | / |
| 0.25% Trypsin-EDTA | HyClone | SH30042.01 | / |
| Agarose | Beyotime | ST118 | / |
| Antibody Dilution Buffer (Primary) | Beyotime | P0023A | / |
| Antibody Dilution Buffer (Secondary) | Beyotime | P0023D | / |
| BCA Protein Assay Kit | Beyotime | P0012 | / |
| BD Cytofix/Cytoperm™ Fixation/Permeabilization Solution Kit | BD Biosciences | 554722 | / |
| BD Pharmingen™ Transcription Factor Buffer Set | BD Biosciences | 562574 | / |
| CellTrace™ Violet Cell Proliferation Kit | Invitrogen | C34858 | / |
| Collagenase Type I | Sigma-Aldrich | SCR103-250mg | / |
| DAPI Staining Solution | AIFang Biological | AFIHC044 | / |
| Deoxyribonuclease I (DNase I) | Sigma-Aldrich | 11284932001 | / |
| DMEM High-Glucose Medium | HyClone | SH30243.01 | / |
| Dynabeads™ Mouse T-Activator CD3/CD28 | Gibco | 11452D | / |
| EasySep™ Mouse CD8a+ T Cell Isolation Kit II | STEMCELL Technologies | 18953 | / |
| EasySep™ Mouse MDSCs Isolation Kit | STEMCELL Technologies | 19867 | / |
| Enhanced Chemiluminescence (ECL) Substrate | Beyotime | P0018S | / |
| Exosome-Depleted FBS Medium | Umibio | UR51102 | / |
| Exosome Extraction Kit | Umibio | UR52101 | / |
| Exosome Purification Column | wisebio. | WSP0015 | / |
| Fetal Bovine Serum (FBS) | Gibco | 10099141C | / |
| FGPL2 Protein, Mouse | MCE | HY-P700990 | / |
| Fixable Viability Dye eFluor™ 780 | Thermo Fisher Scientific | 65-0865-18 | / |
| GW4869 | MCE | HY-P19363-5 | / |
| Leukocyte Activation Cocktail, with BD GolgiPlug™ | BD | 550583 | / |
| Magnetic Bead-based Exosome Isolation Kit | wisebio. | WSR0002-2 | / |
| MDSCs Depletion Agent (Anti-Gr-1) | Bioxcell | BE0075 | / |
| Mouse Fgl2 ELISA Kit | UpingBio | SYP-M0656 | / |
| Multiplex Immunofluorescence Staining Kit | AIFang Biological | AFIHC037 | / |
| Nucleic Acid Stain | Beyotime | D0128 | / |
| Penicillin-Streptomycin Solution (100X) | Gibco | 15140122 | / |
| PKH26 Red Fluorescent Cell Linker Kit | Umibio | UR52302 | / |
| Prestained Protein Molecular Weight Marker | Beyotime | P0078 | / |
| Protease Inhibitor Cocktail | Beyotime | P1005 | / |
| Recombinant Mouse IL-2 Protein | Sino Biological | 51061-MNAE | / |
| Recombinant Mouse GM-CSF Protein | MCE | HY-P7361-10μg | / |
| Recombinant Mouse IL-6 Protein | MCE | HY-P7063-10μg | / |
| RIPA Lysis Buffer | Beyotime | P0013B | / |
| RPMI-1640 Medium | Gibco | C11875500BT | / |
| SDS-PAGE Running Buffer | Beyotime | P0014B | / |
| SDS-PAGE Transfer Buffer | Beyotime | P0575-1L | / |
| Anti-human Calnexin Monoclonal antibody | proteintech | 66903-1-1g | 2A2C6 |
| Anti-human CD63 Monoclonal antibody | proteintech | 67605-1-1g | 3D4D1 |
| Anti-human CD81 Monoclonal antibody | proteintech | 66866-1-1g | 1G2C6 |
| Anti-mouse CD9 Antibody (C-4) | Santa Cruz Biotechnology | sc-13118 | C-4 |
| Anti-human CD9 Monoclonal antibody | proteintech | 60232-1-1g | 4H7B9 |
| sFGL2 protein，Human (HEK293, His-Avi, Flag) | MCE | HY-P700721 |  |
| Anti-mouse HSC70/HSP70 Antibody (W27) | Santa Cruz Biotechnology | sc-24 | W27 |
| Anti-human HSP70 Monoclonal antibody | proteintech | 66183-1-1g | 4E7E |
| Human FGL2 ELISA KIT | UpingBio | SYP-H1022 |  |
| PE Goat anti-mouse IgG (minimal x-reactivity) Antibody | Biolegend | 405307 |  |
| Anti-human TSG101 Monoclonal antibody | proteintech | 67381-1-1g | 2B7G8 |
| Anti-mouse tsg 101 Antibody (Y16J) | Santa Cruz Biotechnology | sc-101254 | Y16J |
| Anti-mouse CD63 Monoclonal Antibody | Abcam | ab134045 | EPR5702 |
| Anti-mouse CD81 Monoclonal Antibody | Abcam | ab79559 | M38 |
| Anti-mouse CD9 Monoclonal Antibody | Abcam | ab236630 | EPR23105-121 |
| Anti-mouse mFgl2 Monoclonal Antibody | R&D Systems | MAB5974-SP | / |
| Anti-mouse sFgl2 Rabbit Monoclonal Antibody | Abcam | ab198029 | / |
| Anti-mouse GAPDH Rabbit Monoclonal Antibody | Abcam | ab8245 | 6C5 |
| β-Actin Rabbit Monoclonal Antibody | Cell Signaling Technology | 4967 | / |
| Anti-mouse Arg-1 (eFluor 450) | Thermo Fisher Scientific | 48-3697-82 | A1exF5 |
| Anti-mouse CD11b (PerCP/Cyanine5.5) | Biolegend | 101228 | M1/70 |
| Anti-mouse CD11c (PE) | Biolegend | 117308 | N418 |
| Anti-mouse CD25 (FITC) | Biolegend | 101907 | 3C7 |
| Anti-mouse CD3ε (Brilliant Violet 421™) | Biolegend | 100227 | 17A2 |
| Anti-mouse CD32B/FcγRIIB (PE) | Invitrogen | 12-0321-82 | AT130-2 |
| Anti-mouse CD4 (BB700) | BD Biosciences | 566407 | RM4-5 |
| Anti-mouse CD44 (Pacific Blue™) | Biolegend | 156006 | NIM-R8 |
| Anti-mouse CD44 (PerCP/Cyanine5.5) | Biolegend | 103032 | IM7 |
| Anti-mouse CD69 (PE) | Biolegend | 985202 | FN50 |
| Anti-mouse CD8a (APC) | BD Biosciences | 553035 | 53-6.7 |
| Anti-mouse CD86 (PE/Cy7) | Biolegend | 159208 | A17199A |
| Anti-mouse CD206 (AF700) | Biolegend | 141734 | C068C2 |
| Anti-mouse Gr-1 (APC) | Biolegend | 108412 | RB6-8C5 |
| Anti-mouse IFN-γ (PE) | Biolegend | 163504 | XMG1.2 |
| Anti-mouse iNOS (PE) | Thermo Fisher Scientific | 12-5920-82 | CXNFT |
| Anti-mouse Ly-6C (AF700) | Biolegend | 108024 | HK1.4 |
| Anti-mouse Ly-6G (PE/Cy7) | Biolegend | 127618 | 1A8 |
| Anti-mouse MHC Class II (I-A/I-E) (Brilliant Violet 510™) | Biolegend | 107636 | M5/114.15.2 |
| Anti-mouse TNF-α (PE/Cy7) | BD Biosciences | 557644 | MP6-XT22 |
| Anti-mouse CD45 (FITC) | Biolegend | 157214 | 30-F11 |
| Anti-Fgl2 mAb (IHC/Neutralizing) | Alpha Diagnostic International | Fgl22-A | / |
| Anti-Human CD11b Monoclonal Antibody | Elabscience | E-AB-60354 | / |
| Anti-Human CD14 Monoclonal Antibody | Elabscience | AN002130P | / |
| Anti-Human CD15 Monoclonal Antibody | Elabscience | E-AB-F11420P | / |
| Anti-Human CD8 Monoclonal Antibody | Cell Signaling Technology | 98941 | D4W2Z |
| Anti-Human CK20 Monoclonal Antibody | Abcam | ab76126 | EPR1622Y |
| Anti-Human FcγRIIB Monoclonal Antibody | Santa Cruz Biotechnology | sc-365864 | / |
| Anti-Rabbit IgG, HRP | Abcam | ab205718 | / |
| Anti-Mouse IgG, HRP | Abcam | ab6728 | / |

**Methods**

**Human sample**

In this study, peripheral blood samples were collected from lung cancer patients in the Department of Oncology at Xinqiao Hospital, as well as from healthy volunteers. Serum exosomes were subsequently isolated and subjected to ELISA, Western blot, and functional assays involving MDSCs. This study was approved by the Medical Ethics Committee of the Second Affiliated Hospital of Army Medical University, PLA (Approval No. Research 2025-231-01). All participants, including healthy volunteers, provided written informed consent prior to sample collection.

**Colon Cancer Patient Tissue Microarray (TMA) Samples**
A tissue microarray (TMA) containing 80 colon cancer samples was procured from Aifang Biotechnology Co., Ltd. The use of these patient samples was approved by the Biological Science and Technology Ethics Committee of Hunan Aifang Biotechnology Co., Ltd. (Approval No.: HN20250401). Written informed consent was obtained from all patients, and the study was conducted in accordance with the principles of the Declaration of Helsinki. The baseline clinical characteristics of the 80 colon cancer patients are summarized in Table S2.

**Multiplex Immunofluorescence (mIF) Staining of Colon Cancer TMA**
TMA sections were routinely deparaffinized in xylene and rehydrated through a graded ethanol series. Multiplex immunofluorescence (mIF) staining was performed using a 7-color multiplex immunofluorescence kit. Antigen retrieval was conducted via microwave treatment according to the manufacturer's instructions. Endogenous peroxidase activity was blocked by incubation with 3% hydrogen peroxide solution at room temperature (RT) for 15 minutes, followed by blocking with 10% goat serum for 15 minutes. The first primary antibody (Primary Antibody A) was applied and incubated overnight at 4°C. After three washes with PBST, a Polymer-HRP anti-mouse/rabbit universal secondary antibody was applied and incubated for 30 minutes at RT. Following PBST washes, Tyramide Signal Amplification (TSA) fluorescence dye was applied for 8 minutes, and sections were washed again three times with PBST. Antibody stripping was performed using microwave treatment to remove Primary Antibody A. After three PBST washes, the blocking step was repeated before applying the next primary antibody (Primary Antibody B). This cycle was repeated sequentially until all target antigens (CK20, Fgl2, FcγRIIB, CD11b, CD14, CD15, CD8) were stained. Finally, sections were counterstained with DAPI solution, incubated for 10 minutes at RT protected from light, washed three times with PBST, and mounted with anti-fade mounting medium. mIF images were acquired using an 8-channel fluorescent digital slide scanner.

**Image Acquisition and Quantification**
During image acquisition, mIF slides were scanned using the 8-channel fluorescent digital slide scanner, and the resulting images were saved in the .kfbf format. For data analysis, a custom algorithm was developed within the VISIOPHARM software platform to analyze the images. To ensure consistency, fixed and uniform threshold parameters were set using the VISIOPHARM application to identify and quantitatively analyze the fluorescence expression of CK20, Fgl2, FcγRIIB, CD11b, CD14, CD15, and CD8. The analysis results were exported upon completion.

**Cell Lines and Culture Conditions**
The MC38 murine colon carcinoma cell line and the Lewis lung adenocarcinoma cell line were utilized for in vitro experiments, exosome isolation, and establishing subcutaneous xenograft models. Both cell lines were purchased from Bohui Biotechnology Co., Ltd. (Shanghai, China). Cells were cultured in DMEM high-glucose complete medium, supplemented with 10% fetal bovine serum (FBS) and 1% penicillin-streptomycin, at 37°C in a humidified incubator with 5% CO₂. Cells were passaged every 2-3 days based on growth characteristics. To prevent mycoplasma contamination, cells were routinely tested monthly using a mycoplasma detection kit to ensure they remained mycoplasma-free and in good condition for subsequent experiments.

**Animal Models**
Female C57BL/6J mice and female FcγRIIB knockout (KO) mice were used to establish animal models. The FcγRIIB KO mice were originally obtained from the laboratory of Professor Wen-Yue Xu (Department of Basic Sciences, our institution). Wild-type (WT) female C57BL/6J mice were purchased from Beijing Vital River Laboratory Animal Technology Co., Ltd. Sample size determination was based on considerations of experimental objectives, expected effect size, variability, and statistical power to ensure the results were statistically significant and reliable. All animal studies were conducted in a non-blinded manner. Mice were housed under specific pathogen-free (SPF) conditions, and their care and experimental procedures strictly followed the guidelines of the Animal Ethics and Use Committee of Army Medical University. All animal experiments were approved by the Animal Ethics and Use Committee of Army Medical University **(Approval No. AMUWEC20210838).**

**Subcutaneous Xenograft Model Establishment.** One day prior to tumor cell inoculation, the hair on the right posterior flank of the mice was removed using an electric shaver. Tumor cells in good condition were harvested by trypsinization, centrifuged, and resuspended in sterile PBS to a final concentration of 1×10⁷ cells/mL. Each mouse received a subcutaneous injection of 100 μL cell suspension (containing 1×10⁶ cells) into the right posterior flank, designated as Day 0. Tumor growth was monitored regularly, and tumor volume was measured every three days using the formula: V = (length × width²) / 2. Mice were euthanized at specific time points after treatment conclusion, and tumor tissues and spleens were collected for analysis.

**Treatment Protocols.** Treatment commenced approximately on day 10 post-inoculation when tumor volumes reached ~100 mm³. The anti-Fgl2 monoclonal antibody treatment group received intraperitoneal (i.p.) injections of 20 μg/kg every two days for a total of four doses. The anti-PD-L1 monoclonal antibody group received i.p. injections of 200 μg per dose every two days for four doses. For the GW4869 (exosome inhibitor) treatment group, a stock solution of GW4869 was prepared at 100 mg/mL in DMSO, aliquoted (50 μL/tube), and stored at -80°C. Before use, the stock was diluted in sterile PBS and administered i.p. at a dose of 2.5 mg/kg/day; an equivalent volume of PBS served as the control. Injections were given every two days for four doses. For the MDSCs depletion group, treatment started on the day of inoculation using an anti-Gr-1 antibody administered i.p. at 200 μg per dose every two days for a total of nine doses.

**Adoptive Transfer Experiments.** Adoptive transfers via tail vein injection also began when tumor volumes reached approximately 100 mm³ (around day 10). The recombinant mouse Fgl2 (rm-Fgl2) protein group received 10 ng per transfer every two days for four doses. The MC38 tumor-derived exosome group received transfers containing 200 μg total exosomal protein every two days for four doses.

**Single-Cell Suspension Preparation and Flow Cytometry Analysis**

**Single-Cell Suspension Preparation.** For tumor tissues, subcutaneous tumors were surgically dissected, minced, and placed in RPMI-1640 digestion solution containing collagenase type I (1 mg/mL) and DNase I (0.1 mg/mL). Tissues were digested for 30 minutes at 37°C with shaking at 120 rpm. The digested tissue was then mashed through a 70 μm cell strainer, centrifuged (2000 rpm, 4°C, 5 min), and the pellet was resuspended in PBS containing 2% FBS. Spleens were washed with PBS, mashed through a 70 μm strainer, subjected to red blood cell (RBC) lysis (1 mL lysis buffer per spleen, 3 min), centrifuged, washed, and finally resuspended in PBS containing 2% FBS.

**Flow Cytometry Surface Staining.** Prior to staining, cells were incubated with anti-mouse CD16/32 antibody (0.25 μg per 100 μL cells) for 20 minutes at 4°C to block Fc receptors. Antibodies were then added at predetermined ratios (viability dye 1:1000, surface antibodies 1:100) and incubated for 30 minutes at 4°C protected from light. Cells were washed three times with FACS buffer (D-PBS supplemented with 2% FBS) and finally resuspended in 200 μL buffer for acquisition.

**Flow Cytometry Intracellular Staining.** After completing surface staining and washing, cells were resuspended in 100 μL of 1× BD Transcription Factor Buffer Set fixative/perm buffer per well and incubated for 30 minutes at 4°C in the dark. Cells were then washed three times with 1× BD Perm/Wash Buffer, resuspended in 100 μL of intracellular antibody diluted 1:100 in Perm/Wash Buffer, and incubated for 30 minutes at 4°C in the dark. Following further washes, cells were resuspended in 200 μL FACS buffer for acquisition.

Details regarding the specific targets and catalog numbers of all flow cytometry antibodies used are provided in Supplementary Table 1. Flow cytometry data were acquired using either a Beckman Coulter Gallios or a BD FACS Celesta flow cytometer and analyzed using FlowJo software (version 10.0).

**In Vitro Induction of MDSCs from Mouse Bone Marrow**
Femora and tibiae were aseptically isolated from 6-week-old female C57BL/6J or FcγRIIB KO mice euthanized by CO₂ asphyxiation and surface-sterilized with 75% ethanol. Surrounding muscle tissue was removed, and bones were rinsed with PBS. Bone ends were cut, and the marrow was flushed out using a syringe and PBS until the bones appeared white. The bone marrow suspension was passed through a 70 μm strainer. After centrifugation, the pellet was treated with 1 mL RBC lysis buffer for 3 minutes, neutralized with PBS, and centrifuged again. The cell pellet was resuspended in complete medium containing recombinant mouse GM-CSF (40 ng/mL) and recombinant mouse IL-6 (40 ng/mL), adjusted to a density of 0.5×10⁶ cells/mL, and plated in 12-well plates (1 mL/well). Cells were cultured at 37°C with 5% CO₂. Fresh cytokine-containing medium was replaced every two days. Induced MDSCs were harvested after 5 days of culture for subsequent experiments.

**Isolation of MDSCs using EasySep™ Mouse MDSCs (CD11b^+^Gr-1^+^) Isolation Kit**
Mice were euthanized by CO₂ asphyxiation and sterilized with 75% ethanol. Spleens were harvested aseptically, washed with ice-cold PBS, and mashed through a 70 μm strainer to prepare single-cell suspensions. After centrifugation, RBCs were lysed using 1 mL lysis buffer for 3 minutes, followed by PBS addition and centrifugation. The pellet was resuspended in sorting buffer (D-PBS without Ca²⁺/Mg²⁺, supplemented with 2% FBS and 1 mM EDTA), filtered, and transferred to a flow cytometry tube. FcR blocking reagent (final concentration 40 µL/mL) and the MDSCs Isolation Cocktail (50 µL/mL) were added sequentially and incubated at RT for 10 minutes. Pre-mixed RapidSpheres™ magnetic particles (75 µL/mL) were added and incubated for 5 minutes at RT. Sorting buffer was added to bring the sample volume to approximately 2.5 mL. The tube was gently mixed and placed in the magnet for 3 minutes. The supernatant containing unlabeled cells was decanted. The magnetically labeled MDSCs were collected, centrifuged, and resuspended for counting. To assess sorting efficiency, an aliquot of 10⁶ cells was stained with anti-CD11b-PE/Cy5.5 and anti-Gr-1-APC antibodies for flow cytometric analysis to determine the percentage and absolute count of MDSCs (CD11b+Gr-1+).

**Isolation of CD8⁺ T Cells using EasySep™ Mouse CD8a Positive Selection Kit II**
Spleens, bilateral inguinal lymph nodes, axillary lymph nodes, and mesenteric lymph nodes were aseptically harvested from healthy 6-week-old female wild-type C57BL/6J mice. Single-cell suspensions were prepared by mashing tissues through a 70 μm strainer. After RBC lysis, the cell pellet was resuspended in 1 mL sorting buffer (D-PBS with 2% FBS and 1 mM EDTA), filtered, and 4 μL FcR Blocking Reagent was added. Components A and B (10 μL each) were pre-mixed, incubated at RT for 5 minutes, then added to the sample and incubated for 3 minutes at RT. RapidSpheres™ magnetic particles (20 μL) were added and incubated for 3 minutes. Sorting buffer (1.5 mL) was added to bring the total volume to ~2.5 mL. The tube was placed in the magnet, and the supernatant was discarded. This wash step within the magnet was repeated three times to enrich the magnetically labeled CD8⁺ T cells positively. Cells were finally resuspended in 1 mL D-PBS (to avoid serum interference for subsequent CTV staining) and counted. Purity and yield were assessed by flow cytometry using an anti-CD8a-APC antibody. For proliferation assays, cells were stained with 5 μM Cell Trace Violet (CTV) for 8 minutes at 37°C, quenched with complete medium, washed, and resuspended at 1×10⁷ cells/mL in RPMI-1640 complete medium.

**MDSCs and CD8⁺ T Cell Co-culture Assay**
CD8⁺ T cells were stimulated using anti-CD3/CD28 activation beads. Beads were washed with D-PBS and resuspended in RPMI-1640 complete medium containing 30 U/mL recombinant mouse IL-2. Beads were mixed with the isolated CD8⁺ T cells at a 1:1 ratio (bead:cell). CD8⁺ T cells (2×10⁵ per well) were plated in 96-well round-bottom plates. MDSCs were added at various ratios (CD8⁺ T : MDSCs = 1:1, 1:2, 1:4, 1:8, 1:16). The total volume was adjusted to 300 μL with complete medium containing an equivalent concentration of IL-2. Co-cultures were maintained for 72 hours at 37°C with 5% CO₂; fresh medium was replenished as needed if significant acidification occurred. For experiments involving MDSCs pre-treated with tumor-derived exosomes, MDSCs (5×10⁵/mL, 1 mL) isolated from tumor-bearing mice were co-incubated with MC38 tumor-derived exosomes (80 μg/mL) in a 24-well plate for 24 hours. MDSCs were then harvested, recounted (including gentle trypsinization if adherent), and co-cultured with activated CD8⁺ T cells at different ratios (MDSCs : CD8⁺ T = 1:4, 1:8, 1:16) for 72 hours. CD8⁺ T cell proliferation was assessed by flow cytometry (CTV dilution).

**Western Blotting (WB)**
Tumor cells were lysed on ice using RIPA lysis buffer for 15 minutes, followed by brief sonication. Lysates were centrifuged at 14,000 × g for 10 minutes at 4°C, and the supernatant was aliquoted and stored. Protein concentration was determined using the BCA assay: a standard curve was generated using BSA, diluted samples were incubated with the working reagent at 37°C for 30 minutes, and absorbance was measured at 562 nm. Prior to electrophoresis, protein samples were mixed with 5× Laemmli loading buffer and denatured at 95°C for 10 minutes. Proteins (20 μg per lane) were separated by SDS-PAGE (80 V through stacking gel, 120 V through resolving gel). Proteins were transferred to activated PVDF membranes using a wet transfer system at 250 mA for 100 minutes in an ice bath. Membranes were blocked with rapid blocking buffer for 1 hour, washed with TBST, and incubated sequentially with primary antibodies overnight at 4°C and HRP-conjugated secondary antibodies for 1 hour at RT. After thorough washing with TBST, bands were visualized using ECL substrate and imaged.

**Tumor Cell and MDSCs Co-culture**
MDSCs were first isolated magnetically from the spleens of wild-type tumor-bearing mice and counted. MC38 tumor cells in good condition and logarithmic growth phase were harvested by trypsinization, centrifuged, resuspended, and counted. Tumor cells (5×10⁵) were placed in the upper chamber of a 24-well transwell insert with a 1.0 μm pore size. An equal number of MDSCs (5×10⁵) were plated in the bottom well of the 24-well plate. The insert containing tumor cells was placed into the well, and complete medium was gently added to a final volume of 1 mL. Different treatments were applied based on experimental groups: Blank Control (only MDSCs in lower chamber), Control (co-culture without treatment), recombinant mouse Fgl2 protein group (20 ng/mL), and Fgl2 monoclonal antibody treatment group. Each group had six replicates. After 48 hours of co-culture, three replicates per group were randomly selected for flow cytometric analysis of MDSCs functional markers. MDSCs from the remaining three replicates were subjected to standard RNA sequencing (transcriptome analysis).

**Co-incubation of Recombinant Mouse sFgl2 Protein with MDSCs**
Recombinant mouse Fgl2 protein was co-cultured with different types or sources of MDSCs. Splenic MDSCs were magnetically isolated from wild-type C57BL/6J tumor-bearing mice and FcγRIIB KO tumor-bearing mice (as in Section 2.8). Bone marrow-derived MDSCs (BMDSCs) were induced from wild-type healthy mice as described in Section 2.7. MDSCs were plated at 2×10⁵ cells per well in flat-bottom 96-well plates. Complete medium containing recombinant mouse Fgl2 protein at various concentrations (0, 20, 40, 80, 100 ng/mL) was added to a final volume of 300 μL per well.

**Co-incubation of Tumor-Derived Exosomes with MDSCs**
To investigate the effect of tumor-derived exosomes on MDSCs immunosuppressive function, MDSCs isolated from the spleens of tumor-bearing mice were co-incubated with exosomes at different concentrations (0, 20, 40, 80, 120, 160 μg/mL) for 24 hours. Expression levels of Arg-1 and iNOS in MDSCs were assessed by flow cytometry. To specifically verify the role of exosomal Fgl2, exosomes were pre-treated with a mouse Fgl2 monoclonal antibody (final concentration 1 μg/mL) for 1 hour at 4°C to neutralize Fgl2 activity before co-incubation with MDSCs for 24 hours. Control groups included MDSCs alone (no exosomes) and MDSCs co-incubated with untreated exosomes. Expression levels of Arg-1 and iNOS in MDSCs were analyzed by flow cytometry.

**Isolation and Purification of Exosomes from Tumor Cell Culture Supernatant**
Tumor cells in good condition were grown until ~50% confluency, at which point the medium was replaced with a 50:50 mixture of exosome-depleted specific medium and complete medium. When cells reached 70–80% confluency, the medium was discarded, and cells were washed 2–3 times with PBS. Cells were then cultured in serum-free exosome-specific medium for 24–48 hours until confluency exceeded 90%. The conditioned supernatant was collected for exosome extraction. The supernatant was centrifuged at 300 × g for 10 min (4°C) to remove live cells, 2000 × g for 10 min (4°C) to remove dead cells, and 10,000 × g for 10 min (4°C) to remove cell debris. The supernatant was sequentially filtered through 45 μm and 22 μm filters. The filtrate was transferred to a 50 mL ultrafiltration concentrator (30 kDa MWCO) and centrifuged at 3000 × g for 8 min (4°C) until concentrated to ~2 mL. Exosome Concentration Solution (ECS) was added to the concentrate at a 4:1 ratio (ECS: concentrate), vortexed, and incubated at 4°C for 8 hours. The mixture was then centrifuged at 10,000 × g for 60 min (4°C). The supernatant was discarded, and the pellet was resuspended in ice-cold PBS. Insoluble material was removed by centrifugation at 12,000 × g for 2 min (4°C), and the supernatant containing exosomes was collected. Further purification was performed using Size Exclusion Chromatography (SEC): 500 μL of sample was loaded onto an exosome purification column and centrifuged at 1000 × g for 2 min (4°C). The flow-through containing purified exosomes was collected, aliquoted, and stored at -80°C. All procedures were performed under a biological safety cabinet using sterile reagents and consumables.

**Isolation of Exosomes from Mouse Peripheral Blood**
Blood was collected via cardiac puncture. deeply anesthetized mice were fixed in a supine position, and the chest skin was disinfected. A 25G needle attached to a syringe was inserted into the heart at a ~30° angle from the xiphoid process, and approximately 0.5 mL of blood was withdrawn into a serum separator tube. Immediately after blood collection, mice were euthanized by CO₂ overdose. Blood was allowed to clot at 4°C for 5 minutes and then centrifuged at 3000 rpm for 10 minutes to separate serum. Serum was further centrifuged at 2000 × g for 10 min to remove cells and debris, followed by centrifugation at 14,000 × g for 30 min to remove larger particles. The supernatant was collected for exosome isolation. WisMag Exosome Beads V2 magnetic beads (100 μL) were placed on a magnetic stand to remove the storage solution. Serum sample (500 μL; if less, topped up with Solution A) was added and incubated for 30 minutes at RT on a rocking platform (17 rpm). The tube was placed on the magnet, and the supernatant was discarded. The beads were washed twice with Solution A. Finally, 100 μL of Elution Buffer was added, vortexed, and incubated on the rocker for 30 minutes. The tube was placed on the magnet, and the supernatant containing purified exosomes was collected, aliquoted (20 μL/tube), and stored at -80°C.

**Characterization of Tumor-Derived Exosomes by Transmission Electron Microscopy (TEM)**
Exosomes were negatively stained and visualized using carbon-supported copper grids. Briefly, 10–20 μL of exosome suspension was dropped onto a paraffin film. A copper grid with the carbon-coated side facing down was gently placed onto the droplet for 5–10 minutes to allow adsorption. Residual liquid was carefully removed using filter paper. The grid was then fixed with 2% glutaraldehyde for 5 minutes, followed by three washes with ultra-pure water. Subsequently, staining was performed with 1% uranyl acetate (pH 4.0–5.0) for 1–2 minutes protected from light. After washing and air-drying, the grid was examined under a transmission electron microscope. Typical exosome morphology was observed: cup-shaped or round vesicles with a darkly stained periphery and a lighter, electron-lucent center, exhibiting diameters within the characteristic exosome size range.

**Nanoparticle Tracking Analysis (NTA) of Tumor-Derived Exosomes**
A 100 μL aliquot of the tumor-derived exosome sample was diluted 10-fold in sterile PBS and mixed by gentle vortexing for 5–10 seconds to avoid structural damage caused by vigorous agitation. The NTA instrument was started, and the laser was pre-warmed for 10–15 minutes. The system was calibrated using 100 nm standard polystyrene beads. One milliliter of the standard bead suspension or the diluted sample was injected into the sample chamber. Camera focus, sensitivity, and scatter intensity were adjusted (typically between 12–16). Shutter speed was set to 15–30 ms and frame rate to 25–30 frames per second (fps). For each diluted sample, at least three video tracks of 30–60 seconds each were captured. Particle size distribution (30–150 nm) and concentration were analyzed. The original sample concentration was calculated by incorporating the dilution factor. Size distribution graphs, concentration data, and representative particle movement tracks were exported.

**Confocal Microscopy for Observing MDSCs Phagocytosis of Fluorescently Labeled Exosomes**
The protein concentration of the exosome preparation was determined using the BCA assay for quantification. The PKH26 linker stock solution was diluted 10-fold in Diluent C to prepare a 100 μM dye working solution. An appropriate volume of the dye working solution was added to the exosomes based on protein amount (50 μL for 10–200 μg; 100 μL for 200–500 μg), followed by vortexing for 1 minute and incubation in the dark for 10 minutes. Unbound dye was removed using an Exosome Spin Column (MWCO 3000) by centrifugation (1000 × g, 2 minutes, 4°C). The purified fluorescently labeled exosomes were collected, stored at 4°C, and their final concentration was determined. MDSCs, isolated from the spleens of tumor-bearing mice, were seeded into 35 mm glass-bottom dishes at 5×10⁵ cells per well. Fluorescently labeled exosomes were added at concentrations of 40 μg/mL and 160 μg/mL and co-incubated with the MDSCs for 6 hours. Cell nuclei and surface Gr-1 were stained using DAPI and an anti-mouse Gr-1-APC antibody, respectively. The phagocytosis of exosomes by MDSCs was subsequently observed and imaged using a confocal laser scanning microscope.

**Enzyme-Linked Immunosorbent Assay (ELISA)**
A sandwich enzyme-linked immunosorbent assay was employed. Microplates pre-coated with an anti-mouse Fgl2 capture antibody were used. Mouse Fgl2 standards and test samples (tumor-derived exosomes, exosome-depleted serum from tumor-bearing mice, exosome-depleted cell culture supernatants, etc.) were added to the wells, followed by the addition of a biotin-conjugated detection antibody. After incubation and thorough washing, horseradish peroxidase (HRP)-conjugated streptavidin was added. Following another incubation and washing step to remove unbound components, a solid-phase complex of capture antibody-antigen-biotinylated detection antibody-streptavidin-HRP was formed on the plate. Tetramethylbenzidine (TMB) substrate was added, yielding a blue product. The reaction was stopped with stop solution, converting the color to yellow. The absorbance (Optical Density, OD) was measured at 450 nm using a microplate reader. The OD value is directly proportional to the concentration of mouse Fgl2 in the sample. A standard curve was generated from the standards, and the concentration of Fgl2 in the samples was calculated accordingly.

**Transcriptome Sequencing and Bioinformatic Analysis**
To decipher the transcriptomic mechanisms underlying the regulation of MDSCs immunosuppressive function by the tumor exosome-Fgl2/FcγRIIB signaling axis, we performed transcriptome sequencing and analysis on MDSCs co-cultured with tumor cells (via transwell, non-contact) and control MDSCs. Total RNA was extracted (with RNA Integrity Number, RIN > 8.0), and cDNA libraries were constructed. Sequencing was performed on the Illumina NovaSeq 6000 platform. Raw sequencing data underwent quality control, adapter trimming, and removal of low-quality reads. Clean reads were aligned to the mouse reference genome (GRCm39). Differential gene expression analysis was performed using DESeq2, with genes satisfying |log2FoldChange| > 1 and adjusted p-value < 0.05 considered significantly differentially expressed. Gene Ontology (GO) functional annotation, Cellular Component (CC) analysis, and Kyoto Encyclopedia of Genes and Genomes (KEGG) pathway enrichment analysis were conducted on the differentially expressed genes using the cluster Profiler software (p-value < 0.05 considered significant).

**Table S2. Primer sequences for human samples in RT‑qPCR experiments**

| Primer sequence | sequence (5'-3') |
| --- | --- |
| H-STAT3-F | GGAGAAGGACATCAGCGGTAAGA |
| H-STAT3-R | CCTCCTTGGGAATGTCAGGATAG |
| H-MYC-F | TACAACACCCGAGCAAGGAC |
| H-MYC-R | AGCTAACGTTGAGGGGCATC |
| H-CCND1-F | CCTCGGTGTCCTACTTCAAATGT |
| H-CCND1-R | TTCATCTTAGAGGCCACGAACAT |
| H-BCL2-F | GGATTGTGGCCTTCTTTGAGTTC |
| H-BCL2-R | CTTCAGAGACAGCCAGGAGAAAT |
| H-NFKB1-F | CTGGAACCACGCCTCTAGATATG |
| H-NFKB1-R | CAGCTGTTTCATGTCTCCTTGTG |
| H-IL6-F | AACATGTGTGAAAGCAGCAAAGA |
| H-IL6-R | CTCTGGCTTGTTCCTCACTACTC |
| H-SOCS3-F | TCCAAACAGGGGACACTTCG |
| H-SOCS3-R | GGGGGTGTGACCATTTCCTT |
| H-AKT1-F | CCTCTGCTTTGTCATGGAGTACG |
| H-AKT1-R | AGCCCGAAGTCTGTGATCTTAAT |
| H-MAPK1-F | GTGTTGCAGATCCAGACCATGAT |
| H-MAPK1-R | TGCAGCCTACAGACCAAATATCA |
| H-PTPN11-F | CCGCTCATGACTATACGCTAAGA |
| H-PTPN11-R | TCCATGATGCTCTCCTGCTTATG |
| H-ARG1-F | AGCAAAGAGAAGTGTCAGAGCAT |
| H-ARG1-R | GCAGACCAGCCTTTCTCAATACT |
| H-PTPN6-F | CAGAAGCAGGAGGTGAAGAACTT |
| H-PTPN6-R | CGATGTAGGTCTTAGCGTTCTCA |
| H-NOS2-F | CCAAGCTCTACACCTCCAATGT |
| H-NOS2-R | GCTGGATGTCGGACTTTGTAGAT |

**Table S3. Clinical characteristics of 80 patients with colorectal cancer**

| **Variables** | **N=80（%）** | **Variables** | **N=80（%）** |
| --- | --- | --- | --- |
| **Gender** |  | **Age** |  |
| Male | 44（55） | <60 | 36（45） |
| Female | 36（45） | >60 | 44（55） |
| **T stage** |  | **Clinical stage** |  |
| T1 | 0（0） | Ⅰ | 4（5） |
| T2 | 8（10） | Ⅱ | 37（46.25） |
| T3 | 30（37.5） | Ⅲ | 29（36.25） |
| T4 | 42（52.5） | Ⅳ | 10（12.5） |
| **N stage** |  | **M stage** |  |
| N0 | 45（56.25） | M0 | 69（86.25） |
| N1 | 20（25） | M1 | 11（13.75） |
| N2 | 15（18.75） |  |  |
| **Treatment strategy** |  |  |  |
| pMMR | 37（46.25） |  |  |
| d MMR | 43（53.75） |  |  |


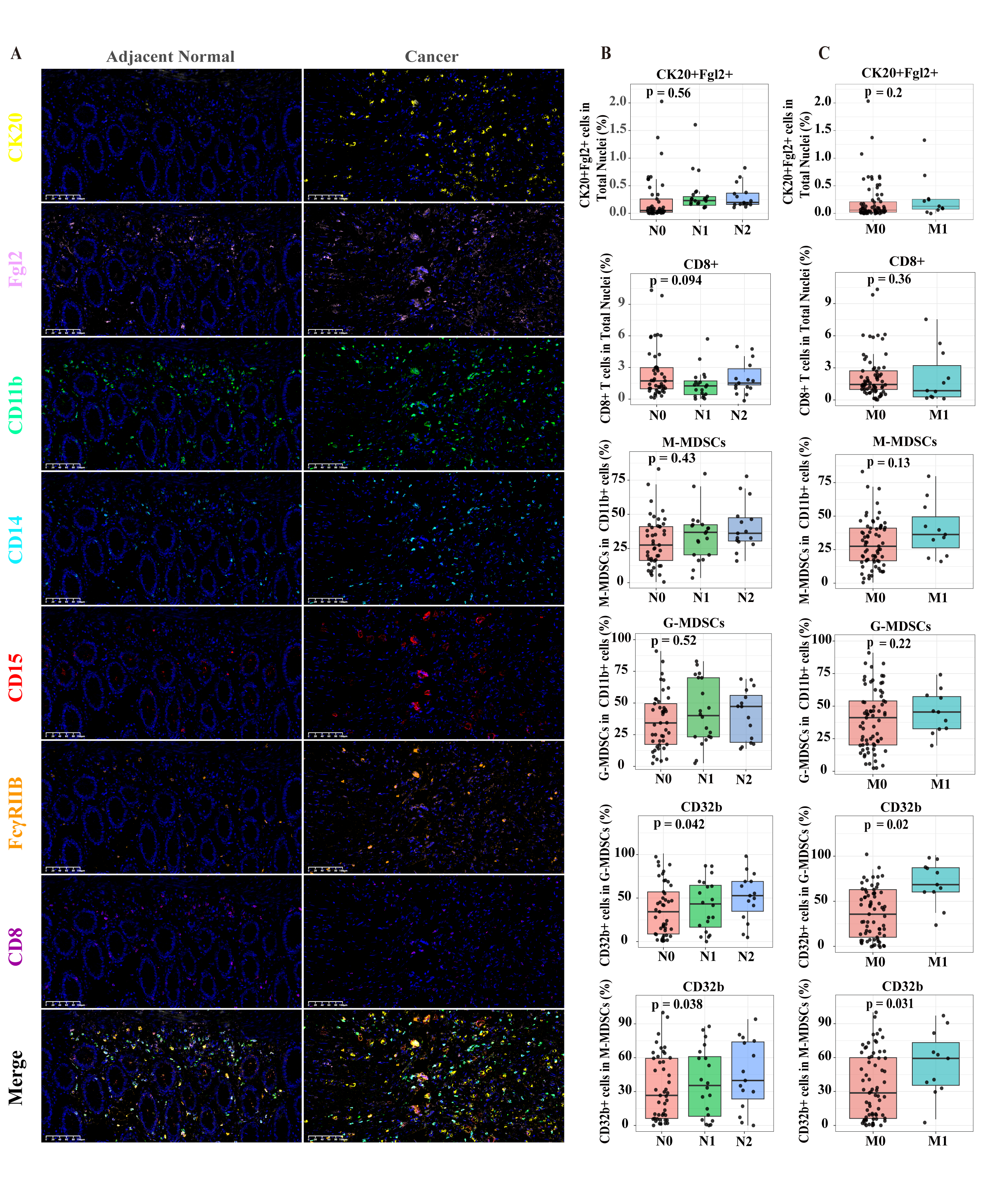


Fig.S1. Analysis of immune cell characteristics in colon cancer tissues and adjacent normal tissues according to N and M staging. (A) Representative multiplex immunofluorescence images of tissue microarrays from 80 colon cancer patients, showing cancerous and adjacent normal tissues. (B) Comparison of the proportion of FGL2⁺ tumor cells, density of CD8⁺ T cells, ratio of M-MDSC to G-MDSC subsets, and expression level of FcγRIIB across N stages. (C) Comparison of the proportion of FGL2⁺ tumor cells, density of CD8⁺ T cells, ratio of M-MDSC to G-MDSC subsets, and expression level of FcγRIIB between M stages. Data are presented as box plots (displaying median, interquartile range, and full range) overlaid with scatter plots. Comparisons among multiple groups for N staging were performed using the Kruskal–Wallis test, while comparisons between two groups for M staging were conducted using the Mann–Whitney U test. Significance levels are defined as follows: *p < 0.05, **p < 0.01, ***p < 0.001, ****p < 0.0001.


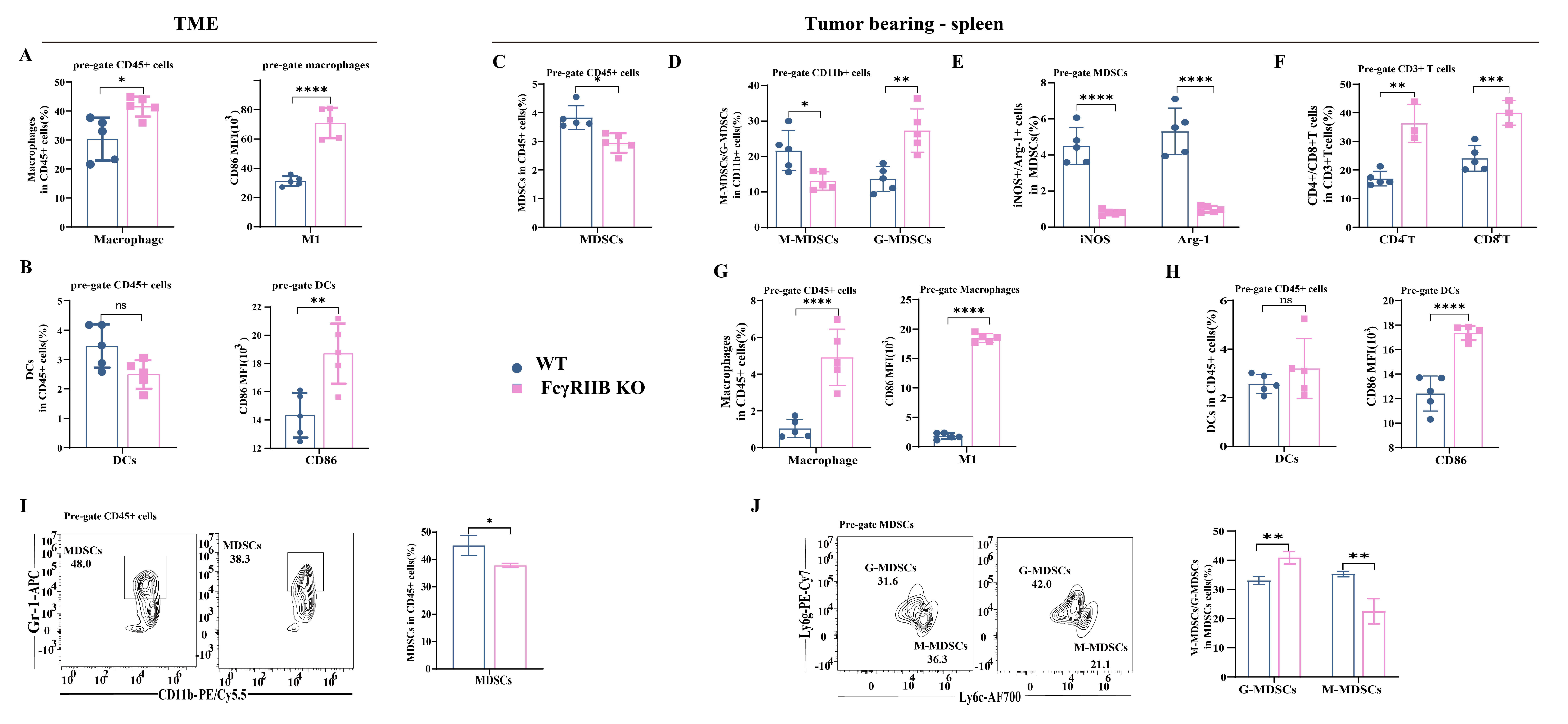


Fig. S2. Systemic and tumor microenvironmental alterations in FcγRIIB KO mice coupled with Fgl2-FcγRIIB functional validation. Tumor microenvironment alterations in FcγRIIB KO mice: (A) Tumor macrophage proportion and M1 macrophage MFI. (B) Tumor DCs infiltration and CD86 MFI on DCs. Systemic immune changes in spleen (flow cytometry): (C) Total MDSCs proportion. (D) G-MDSCs/M-MDSCs ratio. (E) Arg-1 and iNOS expression in MDSCs. (F) CD4⁺/CD8⁺ T cell ratio. (G) Macrophage proportion and M1/M2 polarization ratio. (H) DCs proportion and CD86 expression on DCs. Fgl2-FcγRIIB functional mechanisms: (I-J) In vitro bone marrow MDSCs differentiation and subtype distribution. Animal studies: n = 5 per group; in vitro experiments: n = 3, each performed in triplicate. Data acquired by flow cytometry. Statistical analysis by t-test or one-way ANOVA with Tukey's HSD post-hoc test. Data presented as mean ± SEM. *p < 0.05, **p < 0.01, ***p < 0.001, ****p < 0.0001.


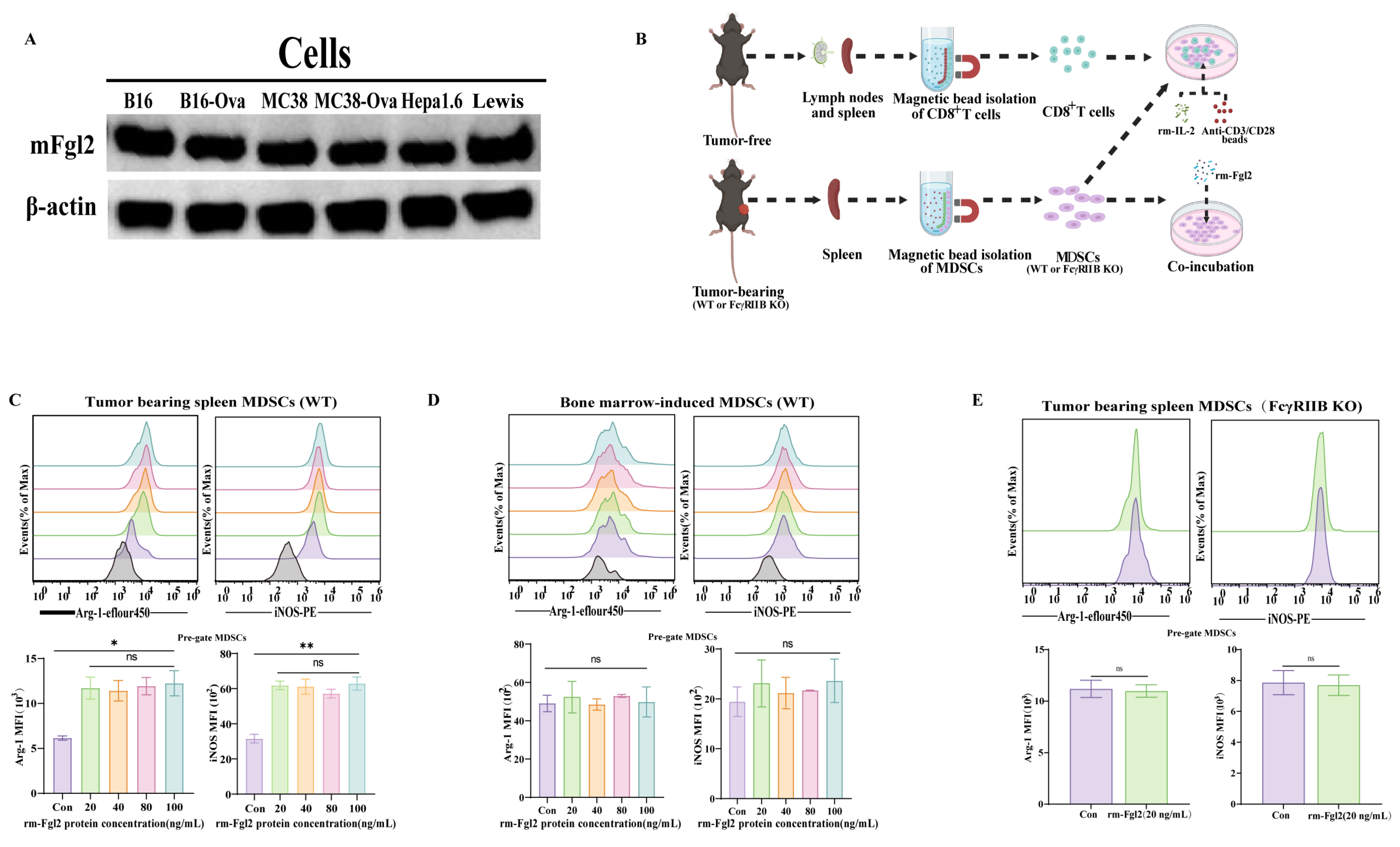


Fig. S3. sFgl2 activates MDSCs in an FcγRIIB-dependent manner. (A)Western blot analysis of mFgl2 protein expression in tumor cells. (B) Schematic of CD8⁺ T cell-MDSCs co-culture and sFgl2 protein incubation with MDSCs, which was created using BioRender.com and for which a publication license has been obtained. (C) Arg-1 and iNOS expression in tumor-bearing mouse MDSCs (high FcγRIIB expression) after sFgl2 treatment. (D) Response to sFgl2 in MDSCs with low FcγRIIB expression (healthy mouse bone marrow-derived). (E) Response of FcγRIIB KO MDSCs to sFgl2 stimulation. In vitro experiments: n = 3, each performed in triplicate. Data acquired by flow cytometry and western blotting. Statistical analysis by one-way ANOVA with Tukey's HSD post-hoc test. Data presented as mean ± SEM. *p < 0.05, **p < 0.01, ***p < 0.001, ****p < 0.0001.


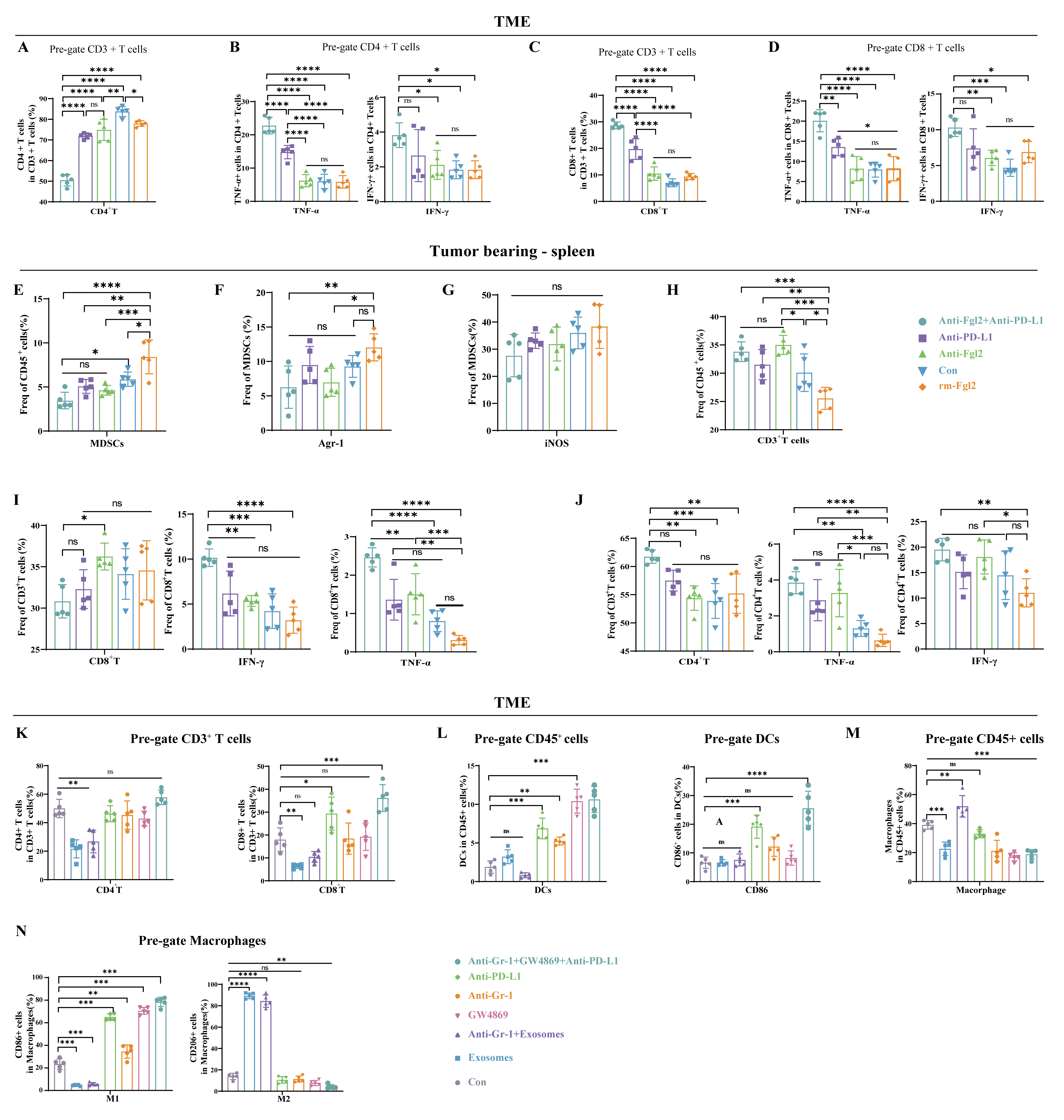


Fig. S4. Systemic immune response and exosomal regulation of MDSCs function. (A-D) Tumor-infiltrating CD4⁺/CD8⁺ T cell proportions and their IFN-γ/TNF-α production. (E) Splenic MDSCs proportions across groups. (F-G) iNOS and Arg-1 expression in splenic MDSCs. (H-J) T cell ratios and cytokine secretion. (K) Tumor-infiltrating CD4⁺/CD8⁺ T cell proportions. (L) Dendritic cell proportion and CD86 expression. (M-N) M1/M2 macrophage polarization ratio. Flow cytometry data: in vivo n=5. Statistical analysis by ANOVA with Tukey's test. Data: mean ± SEM.

*p < 0.05, **p < 0.01, ***p < 0.001, ****p < 0.0001.
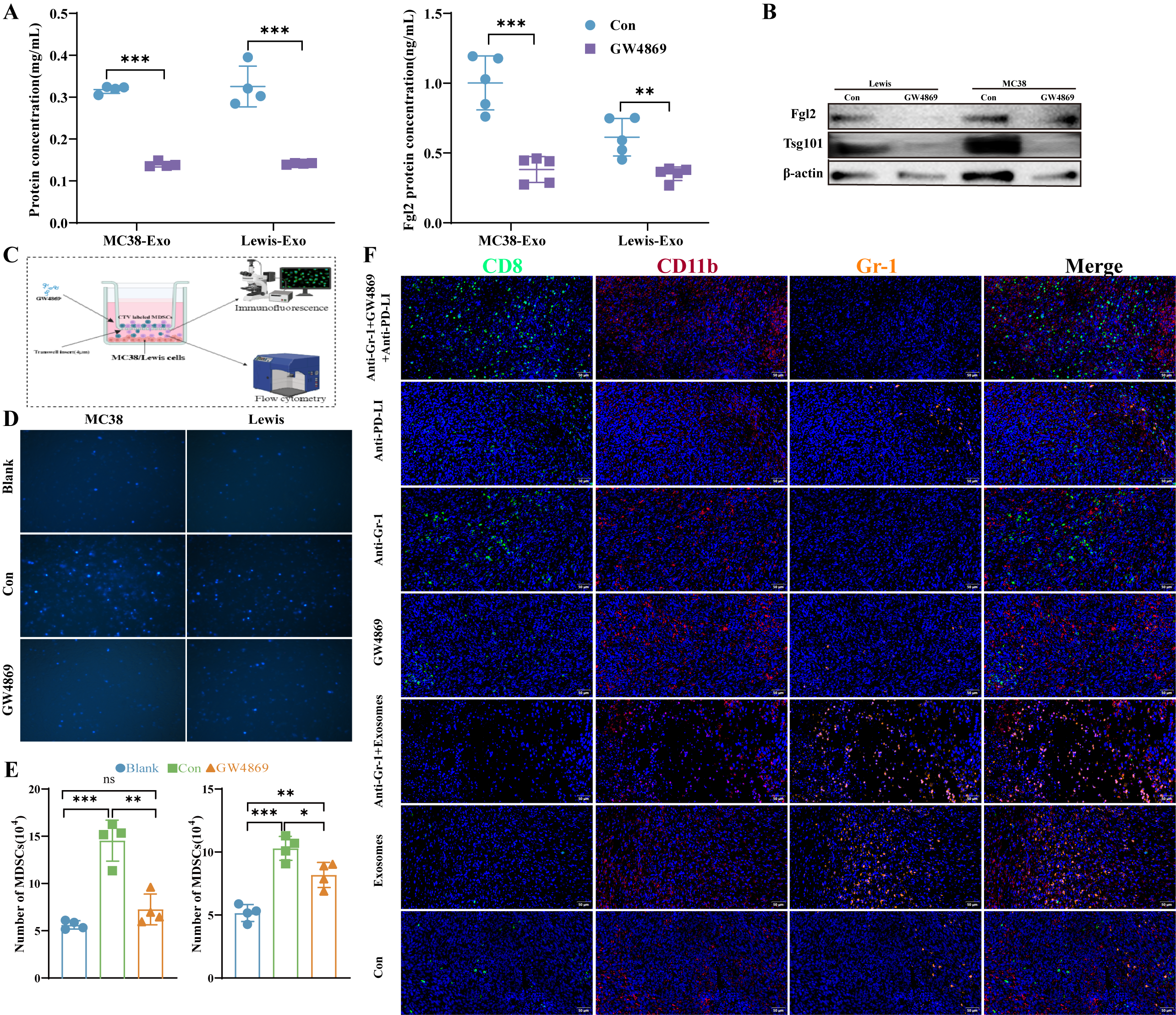


Figure S5. mFgl2-loaded TEX promote MDSCs migration and infiltration. (A) Total protein and mFgl2 protein concentrations in exosomes derived from MC38 and Lewis cell culture supernatants were measured by BCA assay and ELISA, respectively. For each group, supernatants from four 100 cm² dishes were subjected to ultrafiltration and magnetic bead-based exosome extraction, yielding a final volume of 600 μL per sample for subsequent analysis. (B) Western blot analysis of mFgl2 expression in exosomes from MC38 and Lewis cell supernatants. (C) Schematic diagram of the transwell co-culture system with CTV-labeled MDSCs, created by Biorender.com. The upper chamber was seeded with 1 × 10⁶ MDSCs, and the lower chamber of a 12-well plate was seeded with 1 × 10⁶ tumor cells. (D) Microscopic images showing CTV⁺ MDSCs that migrated to the lower chamber. (E) Absolute count of migrated MDSCs collected from the lower chamber, as determined by flow cytometry. (F) Immunofluorescence analysis of MDSC and CD8⁺ T cell infiltration in tumor tissues. Data are shown as mean ± SEM (n = 4 or 5 for in vitro experiments; n = 5 for in vivo experiments). Statistical analysis by t-test or ANOVA with Tukey's test. Data: mean ± SEM. *p < 0.05, **p < 0.01, ***p < 0.001, ****p < 0.0001.


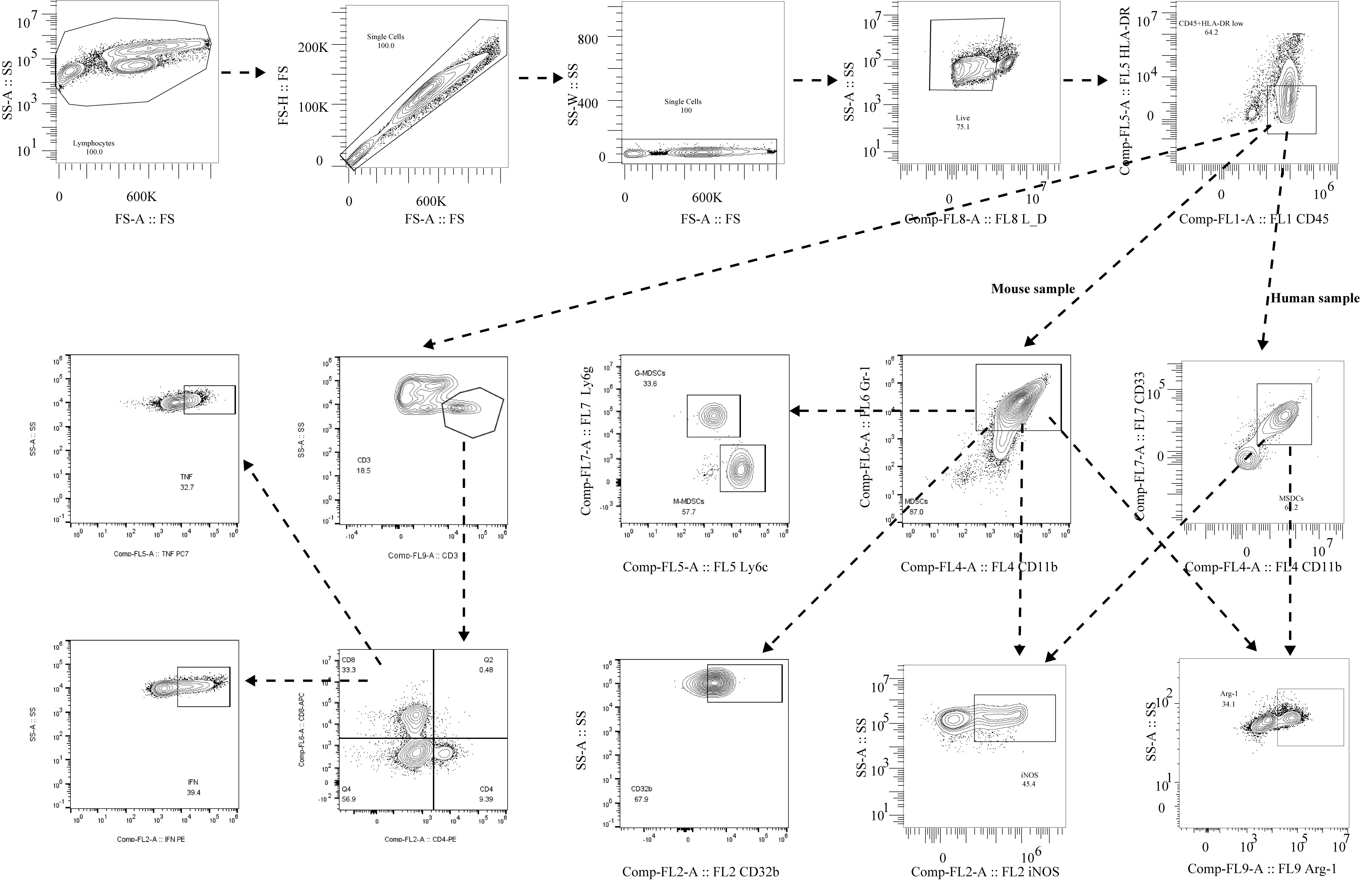


Fig.S6. Flow cytometry gating strategy: Human MDSCs were defined as CD11b⁺CD33⁺HLA-DR^low^, and mouse MDSCs were defined as CD11b⁺Gr-1⁺. Mouse MDSC subsets were identified as M-MDSCs (CD11b⁺Ly6c^high^) and G-MDSCs (CD11b⁺Ly6g^high^). FcγRIIB expression on mouse MDSCs, as well as the functional markers iNOS and Arg-1, were analyzed. T cell subsets, including CD3⁺, CD4⁺, and CD8⁺ T cells, and their functional markers IFN-γ and TNF-α, were also examined. For cell surface protein expression and cytokine expression, our flow cytometry gating strategy is based on isotype or FMO negative controls, such as (iNOS, Arg-1, TNF-α, IFN-γ, FcγRIIB CD32b).


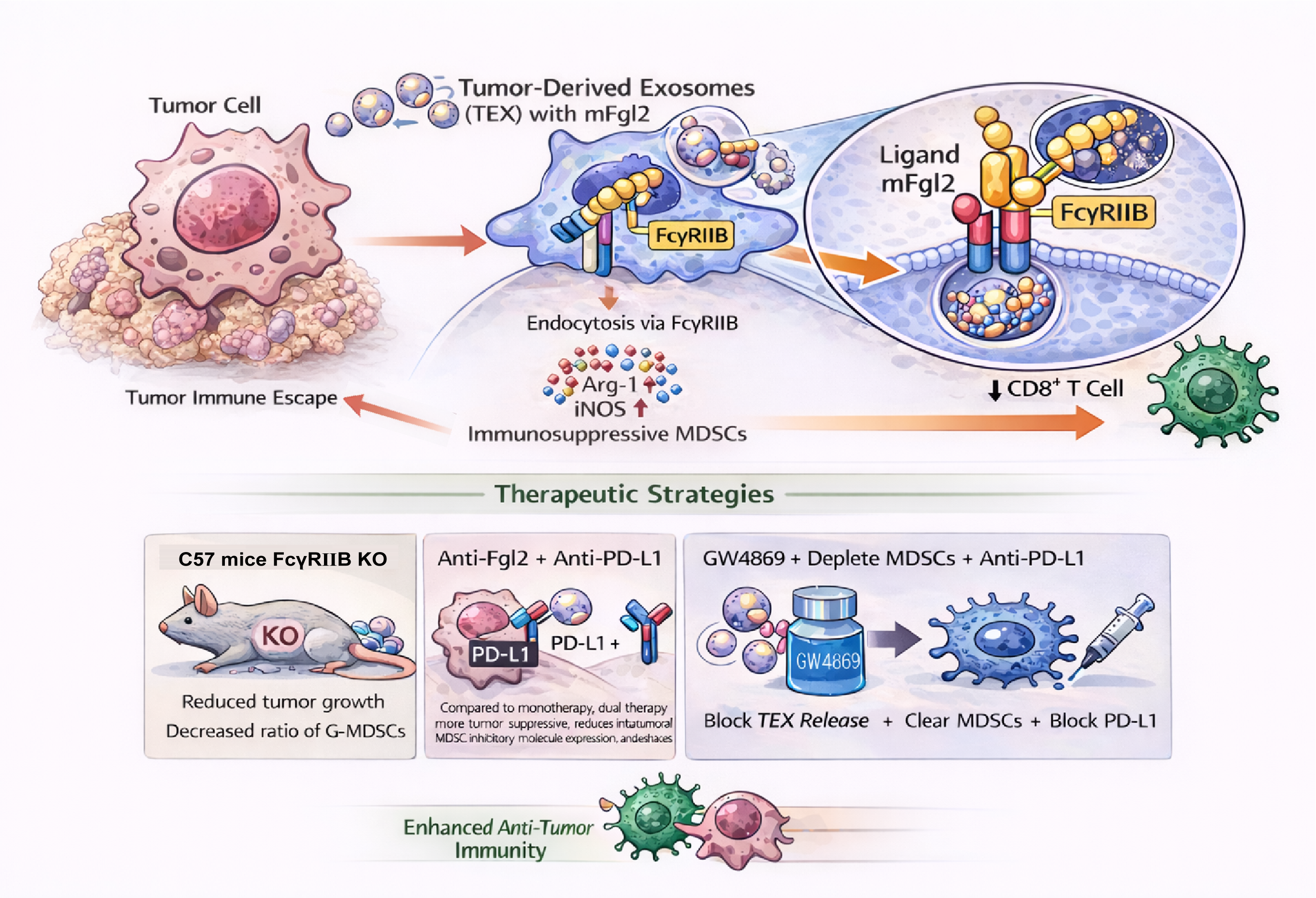


Fig.S7：Mechanism and therapeutic strategy schematic, created with Adobe Illustrator.
